# From Oligo(Phenyleneethynylene) Monomers to Supramolecular Helices: The Role of Intermolecular Interactions in Aggregation

**DOI:** 10.3390/molecules26123530

**Published:** 2021-06-09

**Authors:** Berta Fernández, Zulema Fernández, Emilio Quiñoá, Félix Freire

**Affiliations:** 1 Departamento de Química Física, Universidade de Santiago de Compostela, 15782 Santiago de Compostela, Spain; 2 Centro Singular de Investigación en Química Biolóxica e Materiais Moleculares (CiQUS), Departamento de Química Orgánica, Universidade de Santiago de Compostela, 15782 Santiago de Compostela, Spain; zulema.fernandez@usc.es (Z.F.); emilio.quinoa@usc.es (E.Q.)

**Keywords:** oligo(phenyleneethynylene), monomer aggregation, supramolecular helices, density functional theory and time-dependent density functional theory calculations, geometry optimizations, absorption spectra, electron circular dichroism spectra

## Abstract

Supramolecular helices that arise from the self-assembly of small organic molecules via non-covalent interactions play an important role in the structure and properties of the corresponding materials. Here we study the supramolecular helical aggregation of oligo(phenyleneethynylene) monomers from a theoretical point of view, always guiding the studies with experimentally available data. In this way, by systematically increasing the number of monomer units, optimized n-mer geometries are obtained along with the corresponding absorption and circular dichroism spectra. For the geometry optimizations we use density functional theory together with the B3LYP-D3 functional and the 6–31G** basis set. For obtaining the spectra we resort to time-dependent density functional theory using the CAM-B3LYP functional and the 3–21G basis set. These combinations of density functional and basis set were selected after systematic convergence studies. The theoretical results are analyzed and compared to the experimentally available spectra, observing a good agreement.

## 1. Introduction

Supramolecular helices are obtained through self-assembly of small organic molecules via non-covalent interactions, which will be responsible for their dynamic and reversible character. These interactions determine the aggregate growth mechanism and tune the supramolecular structure. Therefore, taking into account the structure/function relationship, the corresponding materials will have interesting applications in fields such as: catalysis [1,2,3,4,5], nanoelectronics [6,7,8,9,10,11], or pharmaceutics [12,13,14,15], among others. Due to their high interest, a considerable number of experimental studies on supramolecular aggregates have been reported in the literature. It is worth mentioning the work by Meijer et al. [16,17] where the formation mechanisms of several aggregates have been studied in detail and the key role played by non-covalent forces was pointed out.

The structural determination at molecular and intermolecular levels is crucial to understand the properties of the aggregates and is challenging from both the experimental and the computational point of view. Experimental methods like electron circular dichroism (ECD), X-ray diffraction, atomic force microscopy (AFM), infrared (IR) spectroscopy or Raman spectroscopy provide valuable information to study the aggregates, but mostly the information obtained is not enough or not clear enough to solve the tridimensional structure of the aggregate [18,19,20]. Sometimes, even in the optimal experimental conditions and through the combination of several experimental techniques, it is not possible to put all the information together and determine the aggregate morphology. Therefore, computational studies could play a key role in the structural analysis of the supramolecular aggregates, although the large size of the systems under investigation makes the structural and spectroscopic computations challenging. To surpass this limitation, and taking into account the possible lack of accuracy of the different theoretical methods, it is necessary to combine experimental and theoretical approaches. Following this line, Díaz-Cabrera et al. [21] studied the self-assembly of achiral and chiral 1,3,5-triphenylbenzenetricarboxamides. In a similar way, Greciano et al. [22] analyzed the self-assembly of N-annulated perylene bisimides [PBIs], showing the important role of long-range van der Waals and dipole-dipole electrostatic interactions in the aggregation mechanism of compounds that lack H-bonding groups.

Among all supramolecular helices, those formed by building blocks containing π-conjugated cores—perylenebisimides [PBIs] [23,24,25], benzene-1,3,5-tricarboxiamides [BTAs] [26,27,28], oligo(phenyleneethynylene)s [OPEs] [29,30,31,32,33,34,35], *peri*-hexabenzenecoronenes [HBCs] [36,37,38] and others [39]—attract wide interest due to their potential optical and electronic properties. In these molecules the self-assembly is led by π-π interactions in addition to other non-covalent forces. Moreover, the introduction of long alkyl chains ensures good solubility of the building blocks in non-polar solvents and triggers the formation of supramolecular helical aggregates. Regarding this, linear OPEs have drawn much attention not only for their intrinsic supramolecular properties, but also for the possibility of being used in molecular electronic devices [40,41]. Although several studies in the literature use linear achiral OPEs as building blocks for generating supramolecular scaffolds (the most representative ones bearing long alkyl chains on both edges of the OPE building blocks, or even on all the aromatic rings [42,43,44,45]), few chiral examples are found [29,46]. Recently, we have reported a combined experimental-theoretical study on the determination of the aggregation mechanism in a short, chiral and rigid OPE [(*S*)-**1**] [47]. We concluded that the polymerization process of the monomer led to a remarkable aggregation mechanism, where either a thermodynamic aggregate or a kinetically trapped one—both showing opposite supramolecular chiralities—could selectively be obtained just by modifying the aggregation conditions. In this way, while the formation of short *P*-twisted oligomers produced in-plane aggregation to generate brick-like nanostructures, the formation of large helical supramolecular polymers yielded single-chain *M*-type columnar helical aggregates.

**Figure 1:**
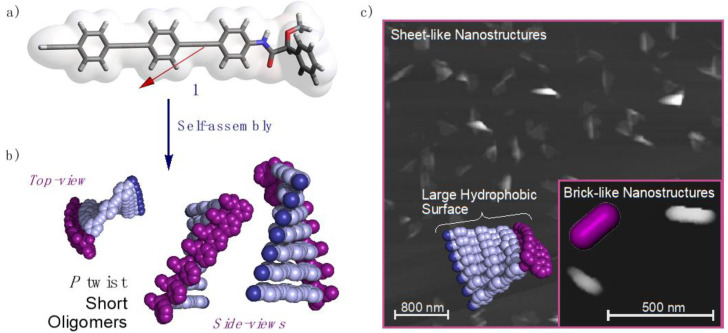
(**a**) Oligo(phenyleneethynylene) (OPE) [(*S*)-**1**] chemical structure, dipole moment and electrostatic potential on the 0.001 au isodensity surface (vdW surface). (**b**) Supramolecular aggregate of (*S*)-**1** describing a *P*-twisted oligomer helix. (**c**) AFM image for the obtained sheet-like nanostructures and inset of the brick-like nanostructures generated by the aggregation of several *P*-twisted oligomer helices.

Herein, we will analyze in detail the aggregation process of (*S*)-**1** (Figure 1a) that results in the formation of sheet-like nanostructures (Figure 1b,c). To perform this investigation the computational studies will be guided with our experimentally available data. First, different morphologies of the aggregate will be generated by stacking the monomers in different orientations and, after determining the optimal one, the number of monomer units in the aggregate will be systematically increased. Computational studies on these systems will allow us to analyze the stability of these aggregates and the intermolecular forces involved in the supramolecular assembly—mainly H-bonding and π-π interactions. Additionally, from the obtained geometries it will be possible to calculate the absorption and circular dichroism spectra. A comparison of these data to the experimentally obtained will be performed looking for the best fit to propose a 3D-model for the aggregate.

The manuscript is organized as follows: in Section 2 we give the computational details, in Section 3 the results are presented and discussed, and in the last Section we summarize and conclude.

## 2. Computational Details

To study the role of intermolecular forces in aggregation, we first analyzed the conformational composition of monomer (*S*)-**1** (n = 1). Once the geometries of the relevant conformers are optimized, we studied a dimer of the optimized monomer (n = 2), which could be built up either with the chiral moieties stacked one on top of the other (head-to-head, hh-geometry) or in an alternate fashion (head-to-tail, ht-geometry). Next, by linking two dimer geometries we created the 4-mers (n = 4) and finally, from the 4-mers, we constructed the 8-mers (n = 8). These sequential additions allowed us to model a helix and study the evolution of its ECD spectrum when the length of the helix is increased. In a previous work, we analysed the ECD spectra of poly(phenylacethylene) (PPA) oligomers [48] by increasing the number of monomer units (n) in the oligomer. From these studies it was concluded that 8–10 monomers were enough to describe the helix generated by the polymers under investigation.

As a first step in the geometry search, for n = 1–4 we carried out molecular dynamics (MD) calculations using the Conformer Rotamer Ensemble Sampling Tool (CREST) program [49] to obtain the most stable and convenient conformers in terms of stacking. The details of the MD simulations are provided in the Supplementary Materials. After selecting the conformers, we further optimized their geometries using Density Functional Theory (DFT) [50,51], together with the B3LYP functional [52,53] with the D3 correction for dispersion [54], and the 6-31G** basis set [55]. We selected this methodology bearing in mind that the main objective of this work is the study of aggregates of monomers where dispersion plays an important role.

To get more insight into the nature of the supramolecular forces, the interaction energies, $E_{A-B}^{\left\{A-B\right\}}$ in Equation (1) were evaluated for the dimer at the DFT(B3LYP-D3)/6–31G** level of theory using the supermolecular approach and the counterpoise method to correct for basis set superposition error [56];

(1) $$\Delta E_{A-B}^{\left\{A-B\right\}}=E_{A-B}^{\left\{A-B\right\}}-E_{B}^{\left\{A-B\right\}}-E_{A}^{\left\{A-B\right\}},$$

where $E_{B}^{\left\{A-B\right\}}$ and $E_{A}^{\left\{A-B\right\}}$ are the energies of monomer *B* and *A*, respectively, and superscript {*A* − *B*} denotes that the energies are evaluated in the dimer basis set.

Additionally, for the most stable conformer of the dimer, in order to elucidate the different contributions to the interaction energy, a non-covalent interaction (NCI) analysis [57] was carried out. The NCI index analysis is based on the density (*ρ*) and its derivatives. The non-covalent interactions can be identified from the reduced density gradient (s) and the sign of the second density Hessian eigenvalue (λ_2_) is used to assign the different interactions. In this way, plots of the variation of s versus (λ_2_ sign) × *ρ* show the nature of the interactions, since this last term can characterize the strength of the interaction through *ρ* and its nature via the λ_2_ sign.

For the 4-mer we obtained the interaction energy by generalizing the expression in Equation (1). Additionally, two-body contributions and cooperative effects are estimated, using the methodology described in reference [58]. In this way, the two body contributions, $\Delta E^{\left(2\right)},$ are given by

(2) $$\Delta E^{\left(2\right)}=\sum_{i>j}^{4}\Delta E_{ij}=\sum_{i>j}^{4}\left(E_{ij}^{\left\{i-j\right\}}-\sum_{k=i,j}E_{k}^{\left\{i-j\right\}}\right),$$

where *i* and *j* are indices that go over the 4 monomers in the 4-mer, and superscript {*I* − *j*} denotes that the dimer basis set is used for the calculations. The total many-body contributions are given as the difference between the complex interaction energy and the two- body contributions in Equation (2). Determining the size of these contributions is important because, on the one hand, they can constitute a considerable part of the interaction energy, therefore playing an important role in the interaction, and on the other hand, considering that many interaction potentials for large molecules are based only on two-body terms, we can check how good a description like this is in the case of the present complexes.

In addition, for the aggregates under investigation it is known that intermolecular H-bonding plays a key role in their formation and stabilization. The stacking of molecules through a hydrogen-bond-network can cause a significant shortening of the hydrogen-bond donor-acceptor distance and an increase of the hydrogen bond strength, whenever a new molecule is incorporated to the supramolecular aggregate [59]. This phenomenon, known as the cooperative effect, has been widely studied in the context of interaction energies, equilibrium structures and spectra [58]. Considering this, here we have also estimated the cooperative effect, defining it with respect to the interaction between monomer A and monomer B in the ABCD 4-mer aggregate as:

(3) $$\Delta E_{ABCD}^{coop}=\Delta E_{A-BCD}-\Delta E_{AB},$$

being

(4) $$\Delta E_{A-BCD}=E_{ABCD}-E_{A}-E_{BCD}$$

and correcting for basis set superposition error.

To evaluate the absorption and ECD spectra, we considered the size of the supramolecular helical structures under investigation and the results of previous studies on covalently bonded polymers [60] that showed the efficiency of employing Time-Dependent DFT (TD-DFT) [61] together with the CAM-B3LYP density functional [62] and the 3-21G basis set [63]. For n = 1–4 we included 80 excitation energies in the calculations and 20 for the 8-mer. To get more insight into the main spectral bands, we evaluated the electron density differences for the corresponding main transitions at the TD-DFT(CAM-B3LYP)/3-21G level of theory. For the n = 1–4 DFT(B3LYP-D3)/6–31G** optimized structures we also evaluated the variation of the IR spectra at the same level of theory.

Due to the reduced size of the monomer, in this case we additionally performed calculations of the UV and ECD spectra with the correlation consistent valence triple zeta (cc-VTZ) basis set and at the experimental X-ray geometry in order to check for geometry and basis set convergence in our results. Also, to get more insight into the lowest energy absorption band of the UV spectrum we evaluated the corresponding vibronic transitions using the CAM-B3LYP functional with the D3 dispersion correction and the 6–31G** basis set.

The optimized geometries, IR and vibronic spectra were obtained with the Gaussian-16 program [64]; all the UV-Vis and ECD spectra were evaluated with the ORCA program [65]; the analysis of the noncovalent interactions was carried out with the NCIPLOT program [57]. To plot the spectra, we used the GABEDIT program [66], and for the density differences Avogadro [67]. For the UV-Vis and ECD spectra we selected a full width at half height (FWHM) of 20.0 nm and employed gaussian curves for the spectra and an isovalue of 0.0002 in the density differences. Details for the IR and vibronic spectra are provided in the Supplementary Materials. No solvent effects were included in the calculations.

## 3. Results and Discussion

**Figure 2:**
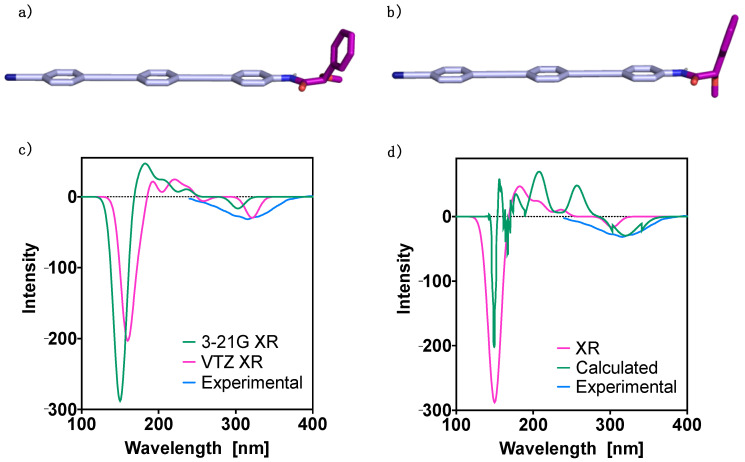
Oligo(phenyleneethynylene) (OPE) [(*S*)-**1**] DFT(B3LYP-D3)/6–31G** optimized geometries for (**a**) the most stable conformer and (**b**) the second most stable conformer. TD-DFT (CAM-B3LYP) ECD spectra: (**c**) 3–21G and cc-pVTZ (VTZ) basis set results for the X-ray (XR) geometries; (**d**) 3–21G basis set results obtained for the X-ray (XR) and the DFT(B3LYP-D3)/6–31G** optimized geometries (the latter denoted Calculated). The corresponding experimental spectra are plotted for comparison. No correction factors are included in the theoretical wavelengths.

**Figure 3:**
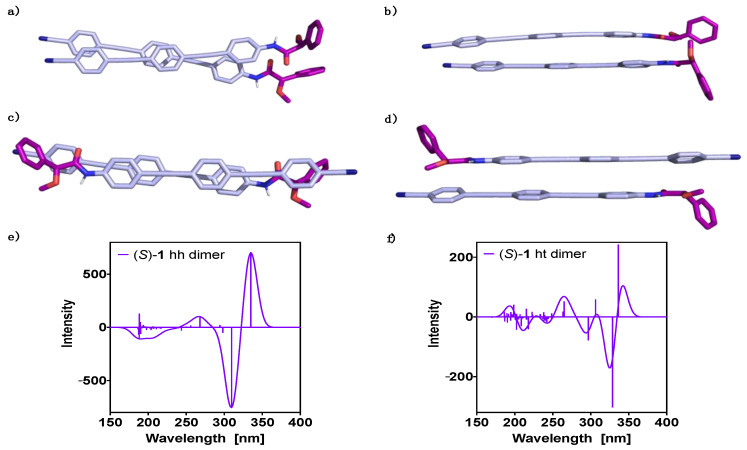
Oligo(phenyleneethynylene) (OPE) (*S*)-**1** dimer DFT(B3LYP-D3)/6–31G** optimized geometries: (**a**) hh perpendicular view, (**b**) hh parallel view, (**c**) ht perpendicular view and (**d**) ht parallel view. TD-DFT (CAM-B3LYP)/3–21G ECD spectra evaluated at the DFT(B3LYP-D3)/6–31G** optimized geometries: (**e**) hh dimer and (**f**) ht dimer. No correction factors are included in the theoretical wavelengths.

**Figure 4:**
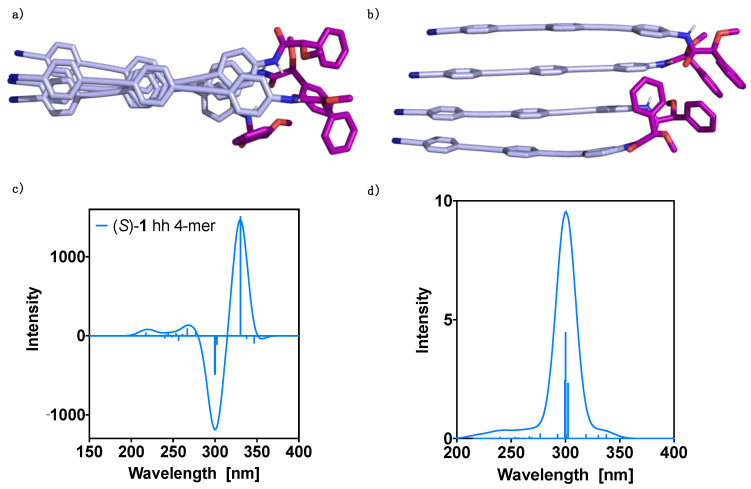
Oligo(phenyleneethynylene) (OPE) (*S*)-**1** hh 4-mer DFT(B3LYP-D3)/6–31G** optimized structure: (**a**) perpendicular view and (**b**) parallel view. TD-DFT (CAM-B3LYP)/3–21G spectra evaluated at the DFT(B3LYP-D3)/6–31G** optimized geometry: (**c**) ECD and (**d**) UV-Vis. No correction factors are included in the theoretical wavelengths.

**Figure 5:**
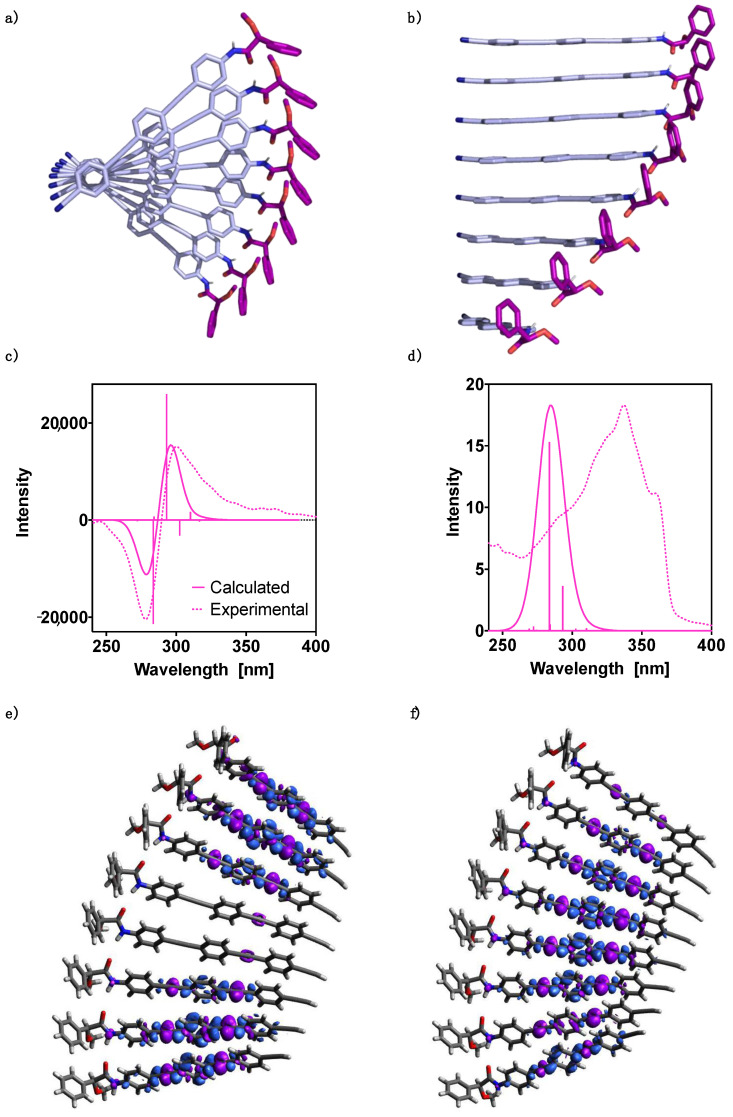
Oligo(phenyleneethynylene) (OPE) (*S*)-**1** hh 8-mer structure: (**a**) perpendicular view and (**b**) hh parallel view. TD-DFT (CAM-B3LYP)/3–21G spectra: (**c**) ECD and (**d**) UV-Vis. The corresponding experimental spectra are plotted for comparison. No correction factors are included in the theoretical wavelengths. Electron density differences (purple color negative and blue positive, isovalue = 0.0002) for transitions: (**e**) S_0_ → S_7_ and (**f**) S_0_ → S_9_.

**Figure 6:**
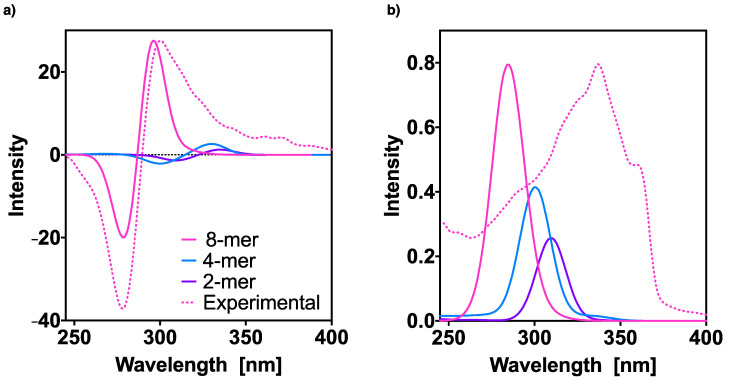
Effect of the aggregation on the TD-DFT (CAM-B3LYP)/3–21G (**a**) ECD and (**b**) UV-Vis spectra of oligo(phenyleneethynylene) (OPE) (*S*)-**1** hh n-mers, n = 2, 4, and 8. The corresponding experimental spectra are included for comparison. No correction factors are considered in the theoretical wavelengths. The 8-mer intensities were normalized with respect to the experimental ones and the 2,4-mer intensities scaled with the same factors.

The obtained results are summarized in Table 1 and Figure 1, Figure 2, Figure 3, Figure 4, Figure 5 and Figure 6. Those for the Molecular Dynamics simulations, the analysis of the noncovalent interactions and the IR and vibronic spectra are provided in the Supplementary Materials.

First theoretical studies were carried out for monomer (*S*)-**1**. The molecule was built up using the geometry determined from X-ray studies [68] as a starting point, and the most stable conformers were obtained using the CREST program. This revealed the presence of two major conformers at room temperature that contribute significantly to the spectra. These contributions are of 70% (degeneracy 58) for the most stable conformer (Conformer 1) and 30% (degeneracy 52) for the second one (Conformer 2). Next, further optimization of these two structures was carried out at the DFT(B3LYP-D3)/6–31G** level, obtaining for Conformer 1 a (O = )C-Cα-O-C(Me) dihedral angle equal to −162° (Figure 2a), while Conformer 2 has a (O = )C-Cα-O-C(Me) dihedral angle equal to −78° (Figure 2b). The energies of these two conformers are shown in Table 1, corresponding to a population of 52.7% for Conformer 1 and of 47.3% for Conformer 2 at room temperature. We also evaluated the populations using the difference in Gibbs free energy, and the corresponding results are reported in the Supplementary Materials (Section 4). Due to the near degeneracy of the two conformers, we additionally carried out the above calculations with the 6311++G(d,p) basis set. The results provided similar conformers in terms of the dihedral angles—(O = )C-Cα-O-C(Me) dihedral angle equal to −164° and −78° for Conformer 1 and 2, respectively—and with an energy difference of 0.49 kcal mol^−1^. For consistency, we include here the 6–31G** values and report the 6311++G(d,p) ones in the Supplementary Materials. Both molecular dynamics and DFT calculations provide Conformer 1 as the most stable.

Next, the theoretical UV-Vis and ECD spectra were evaluated. To perform these studies the TD-DFT method, use of the CAM-B3LYP functional with either the 3-21G [63] or the cc-pVTZ basis set [69], was employed. In a first approach, we evaluated the theoretical ECD spectrum for the geometry obtained from the X-ray studies. Comparison between the two theoretical ECD spectra, calculated using the 3–21G and the cc-pVTZ bases, and the experimental spectrum, is shown in Figure 2c. A good agreement is observed for the first Cotton band in the three ECD spectra. Moreover, in the case of the two theoretical ECD traces, the general shape of the spectra provided by the two bases is very similar, with a displacement of around 30 nm to the right in the case of the cc-pVTZ spectrum.

Then, the ECD spectra for the DFT(B3LYP-D3)/6–31G** optimized geometries of the two conformers with significant population at room temperature were calculated and combined considering the conformer contributions to get the final theoretical ECD trace. A comparison between the ECD traces obtained from the theoretical and the experimental studies, in addition to the theoretical spectrum obtained from the X-ray geometry, is shown in Figure 2d. It was found that both TD-DFT(CAM-B3LYP)/3–21G spectra—the one obtained from the conformational studies and the one obtained from the X-ray geometry—are in agreement with the experimental one in the sign of the first Cotton effect, between 300 and 320 nm. Also, the ECD traces obtained from the theoretical and the X-ray geometries show good agreement around 150 nm, while the intermediate regions of the spectra are considerably different. From the DFT(B3LYP)/6311++G(d,p) conformer geometries we evaluated the corresponding ECD spectrum and report the results in the Supplementary Materials, where it is compared to the 6–31G** one. When considering the populations obtained from the Gibbs free energies, only small differences are observed in the spectral bands, that are mainly due to intensity changes in the high energy regions (see Figure S6 in the Supplementary Materials).

Similar studies were carried out for a dimer of (*S*)-**1** made by the optimized conformers of the monomeric unit. The dimer can be built up in two different ways, head-to-head (hh) (Figure 3a,b) or head-to-tail (ht) (Figure 3c,d). The former (hh) was obtained as the most stable structure, with an energy difference with respect to the latter (ht) of 2.2 kcal mol^−1^ (see Table 1). This large energy gap implies a significant difference in population of the hh with respect to the ht aggregate at room temperature, indicating a negligible contribution of the ht-conformer to the ECD spectra. Additionally, using the counterpoise correction, we evaluated the DFT(B3LYP-D3)/6–31G** interaction energies for the hh and ht dimers, obtaining values of −24.2 kcal mol^−1^ and −21.8 kcal mol^−1^, respectively. This corroborates the larger stability of the hh dimer compared to the ht one. To counterpoise correct the results is important, accounting the corrections to 12.5 kcal mol^−1^ and 10.1 kcal mol^−1^ for the hh and ht dimers, respectively. The results of the analysis of the non-covalent interactions in the hh-dimer are reported in the Supplementary Materials (Figure S4) and show that van der Waals forces clearly dominate the non-covalent interactions. These results are in agreement with the van der Waals surface shown in Figure 1a, defined as that with an electron density equal to 0.001 au, and where the molecular electrostatic potential plotted reflects the electrostatic contribution to possible intermolecular interactions [70].

Theoretical ECD studies—TD-DFT(CAM-B3LYP)/3–21G—for the two orientations of the dimer show ECD traces that agree in the high wavelength region; first (335 nm, positive) and second cotton bands (310–330 nm, negative) (Figure 3e,f). In the low wavelength region (180 to 300 nm) both ECD traces are quite different; thus, while the ht dimer displays many transitions with similar intensities (Figure 3f), the hh dimer shows just a few transitions with significant intensity (Figure 3e).

Considering the higher stability of the hh dimer, we continued studying the formation of higher order aggregates using this geometry. In this way, 4-mers were constructed from the hh dimer optimized geometry and submitted to theoretical structural studies using the CREST program. The obtained conformers were optimized at the DFT(B3LYP-D3)/6–31G** level, resulting in a unique preferred conformer with significant population at room temperature (Figure 4a,b).

The counterpoise corrected interaction energy was calculated at the DFT(B3LYP-D3)/6–31G** level for the 4-mer as the difference between the complex energy and the energies of the corresponding monomers. The value obtained (−91.9 kcal mol^−1^) is lower by 19.3 kcal mol^−1^ than three times the dimer interaction energy, indicating that the formation of the tetramer is favored. Using the methodology outlined in reference [24], two-body contributions and cooperative effects were estimated. The former added up to −91.7 kcal/mol, being therefore the many-body contributions negligible and equal to −0.2 kcal/mol. These results clearly show that it can be a good approximation to include only two-body terms when considering the evaluation of the interaction energies in the aggregates, since these contributions amount for the most important part of the interaction energy in the 4-mer. To get insight into the cooperative effects due to the presence of extra monomer molecules, we calculated them using the same method and basis set and taking as reference the two first monomers in the 4-mer (Equation (3)). A value of −13.5 kcal/mol resulted, showing that they are important at least for the small n-mers.

Subsequently, the ECD and absorption TD-DFT(CAM-B3LYP)/3–21G spectra were calculated and the results are presented in Figure 4c,d, respectively. Thus, the UV-Vis spectrum shows one dominant transition at 300 nm and two secondaries at 299 and 302 nm, resulting in one band centered at ca. 300 nm. On the other hand, the theoretical ECD spectrum shows two intense Cotton bands that govern the spectrum, a first positive Cotton effect at 330 nm and a negative one at about 300 nm. These two bands are considerably more intense than the rest of the spectrum.

Finally, from the optimized 4-mers, 8-mer oligomers were constructed. Due to the large size of the 8-mers, we could not optimize the corresponding structures. As in all steps of the geometry assembly process visual inspection of the conformers was mandatory, in order to select the units that were adequate for an increase in cluster size; hence, often conformers with lack of proper order or monomers located at the edges of the aggregate needed to be disregarded. This resulted in the 8-mer geometry displayed in Figure 5a,b. To get further insight into the cooperative effects, we evaluated for all the n-mers (n = 1–8) of the 8-mer structure the interaction energy per monomer unit and convergence was achieved at around n = 5.

The theoretical 8-mer ECD and UV-Vis TD-DFT(CAM-B3LYP)/3-21G spectra were calculated and compared to the experimental data obtained for an aggregate of (*S*)-**1** (Figure 5c,d, respectively). The theoretical UV-Vis spectrum shows a main band that agrees reasonably well with the experimental one. The dominant intensity at 284 nm is mainly due to a transition to the 9th excited state. Interestingly, both the experimental UV-Vis spectrum of the monomer (Figure S1 in the Supplementary Materials), as well as that of the polymer, show a shoulder in the main band at larger wavelengths. In order to interpret this fact, the vibronic spectrum of the most stable conformer for (*S*)-**1** was evaluated. On this spectrum the influence of the density functional and the basis set differ; when improving the basis set in going from the 3–21G to the 6–31G** one, the spectrum suffers a displacement to larger wavelengths, whereas the opposite scenario occurs when changing the density functional from B3LYP-D3 to CAM-B3LYP-D3. The combination of the latter functional with the 6–31G** basis set provided results that agree well with the experimental data and indicate that the shoulder around 360 nm can be assigned to the |0 > -- > |0 > vibronic transition (see the Supplementary Materials for additional information, Table S1 and Figure S1).

In the case of the theoretical ECD studies, it can be observed that the calculated spectrum is in good agreement with the experimental one (Figure 5c). In both cases a bisignate is obtained, where the two main transitions, at 293 nm with positive intensity and at 284 nm with negative intensity, are due to transitions to the 7th and the 9th excited states, respectively. The calculated electron density differences indicate that electron transfer from the two alkyne intermediate units to the rings in between them takes place in these excitations (Figure 5e,f).

Finally, Figure 6 summarizes all the above results and compares the different aggregate spectra previously obtained for: the dimer, tetramer and octamer of [(*S*)-**1**]_n_ (n = 2, 4, 8). The experimental results for the polymer are also provided. We can observe that as n increases, the spectra undergo a bathochromic shift (to lower wavelengths). The ECD spectrum of the dimer and that of the tetramer show three Cotton effects already with a first positive Cotton band, whereas in the case of the octamer only a bisignate, which perfectly reproduces the experimental spectrum, was obtained.

The IR spectrum results for the n = 1–4 DFT(B3LYP-D3)/6–31G** optimized structures are provided in the Supplementary Materials (Figure S2). Those of the monomer and the dimer display almost the same pattern, showing very small changes in going from the former to the latter. On the contrary, the 4-mer spectrum is considerably different, clearly reflecting the association. In this way, for instance, a displacement of the N-H stretching bars to lower energies indicates the entanglement of the H-bond interactions in the stabilization of the aggregate. This shift is reflected in the presence of an extra band in the 4-mer spectrum, that shows three peaks instead of two in the region around 3300 cm^−1^ (where the terminal ethynyl-H stretching signals are also located). In a similar way, a smaller shift to lower energies of around 20 cm^−1^ is also observed in the case of the amide deformation band located at 1682 cm^−1^ in the monomer. These results corroborate that the aggregates are stabilized by H-bond interactions.

## 4. Summary and Conclusions

Considering the wide interest linear OPEs have attracted due to their supramolecular properties and their possible applications to electronic devices, here we have carried out a detailed structural investigation on the aggregation process of (*S*)-**1**, which yields two sheet-like nanostructures. Through systematically increasing the number of monomer units, we have determined a possible structure for the aggregate and analyzed the intermolecular forces involved in the supramolecular assembly. For the geometry optimizations we used density functional theory together with the B3LYP-D3 functional and the 6–31G** basis set. Additionally, from the obtained geometries we calculated the TD-DFT(CAM-B3LYP)/3–21G absorption and circular dichroism spectra, that are in agreement with the experimental results available. Therefore, we can conclude that the methodology used can be expected to provide an efficient approximation to the structure and spectra in routine studies of similar aggregates and polymers.

## Figures and Tables

**Table 1:** DFT(B3LYP-D3)/6-31G** optimized conformer energies.

		Energy (H)
monomer	Conformer 1	−1476.4953010
	Conformer 2	−1476.4953009
dimer	hh	−2953.0435193
	ht	−2953.0400134
4-mer		
	hh	−5906.1562241

## Supplementary Materials

The following supplementary data are available online. Molecular Dynamics study, Table S1: Molecular Dynamics main results, Table S2: Geometries used in the evaluation of the spectra, Table S3: Monomer vibronic spectrum transitions, Figure S1: MD conformers for the monomer, Figure S2: MD most stable conformers for the dimer, Figure S3: MD most stable conformers for the 4-mer, Figure S4: Analysis of the dimer intermolecular interactions, Figures S5 and S6: Additional ECD spectra for the monomer, Figure S7: Monomer vibronic spectrum, Figure S8: Monomer and 4-mer IR spectra.

## Sample Availability

Samples of the compounds are not available.

## Geometry Availability

The optimized geometries are included in the Supplementary Materials.
