# Supplementary material for: From Oligo(Phenyleneethynylene) Monomers to Supramolecular Helices: The Role of Intermolecular Interactions in Aggregation
Source: Molecules. 2021 Jun 9;26(12):3530. doi: 10.3390/molecules26123530 (PMC8228480; doi:10.3390/molecules26123530)
Supplement: Supplementary file 1 [file molecules-26-03530-s001.zip › molecules-1233535-supplementary.pdf]

molecules

MDPI

Supplementary Materials:

From Oligo(Phenyleneethynylene) Monomers to Supramolecular Helices: The Role of Intermolecular Interactions in Aggregation

Berta Fernández [1,*], Zulema Fernández [2], Emilio Quiñoá [2] and Félix Freire [2,*]

[1] Departamento de Química Física, Universidade de Santiago de Compostela, 15782 Santiago de Compostela, Spain
[2] Centro Singular de Investigación en Química Biolóxica e Materiais Moleculares (CiQUS) and Departamento de Química Orgánica, Universidade de Santiago de Compostela, 15782 Santiago de Compostela, Spain; zulema.fernandez@usc.es (Z.F.); emilio.quinoa@usc.es (E.Q.)
* Correspondence: berta.fernandez@usc.es (B.F.); felix.freire@usc.es (F.F.)

Abstract: Supramolecular helices that arise from the self-assembly of small organic molecules via non-covalent interactions play an important role in the structure and properties of the corresponding materials. Here we study the supramolecular helical aggregation of oligo(phenyleneethynylene) monomers from a theoretical point of view, always guiding the studies with experimentally available data. In this way, by systematically increasing the number of monomer units, optimized n-mer geometries are obtained along with the corresponding absorption and circular dichroism spectra. For the geometry optimizations we use density functional theory together with the B3LYP-D3 functional and the 6–31G** basis set. For obtaining the spectra we resort to time-dependent density functional theory using the CAM-B3LYP functional and the 3–21G basis set. These combinations of density functional and basis set were selected after systematic convergence studies. The theoretical results are analyzed and compared to the experimentally available spectra, observing a good agreement.

Keywords: oligo(phenyleneethynylene); monomer aggregation; supramolecular helices; Density Functional Theory and Time-Dependent Density Functional Theory calculations; geometry optimizations; absorption spectra; electron circular dichroism spectra

Table of Contents

1. Molecular Dynamics study……………………………………………………………………3
2. Table S2: Geometries used in the spectrum calculations…………………………………..8
3. Analysis of the intermolecular interactions……………………………………………..33
4. Monomer additional calculations………………………………………………………………34
5. Table S3: Monomer vibronic spectrum transitions ………………………………………36
6. Figure S7: Monomer vibronic spectrum…………………………………………………….37
7. Figure S8: Experimental and theoretical IR spectra…………………………………….38

1. Molecular Dynamics study

The molecular dynamics (MD) calculations were carried out using the Conformer Rotamer Ensemble Sampling Tool (CREST) program [1], emplying the iMTD-GC conformational search workflow that generates conformer/rotamer ensembles (CREs) through extensive metadynamic sampling, with an additional generic z-matrix crossing (CG) step at the end. We performed the calculations at the GFN2-xTB [2] level and without taking into account solvent effects. For the dimer and the **4-mer** we considered two conformational searches: the first one starting from an hh configuration of the monomers and the second one starting from an ht. In the later case for the dimer we got 50 conformers with relative energies within 6 kcal mol^{-1}, and only the 14th more stable kept an ht configuration, no ht conformer was obtained for the **4-mer**. The results for the most stable conformers are summarized in Table S1 and displayed in Figures S1, S2 and S3. Additional results can be obtained from the authors on request.

Table S1. Most stable conformers obtained in the MD analysis. Results are reported for the monomer and the hh dimer and **4-mer** studies.

**Monomer Set**	**Erel (kcal/mol)**	**weight**	**degeneracy**
1	0.000	0.69583	58
2	0.437	0.29920	52
3	1.184	0.00326	2
4	2.795	0.00164	15
**Dimer Set**	**Erel (kcal/mol)**	**weight**	**degeneracy**
1	0.000	0.88749	234
2	0.718	0.02749	25
3	0.916	0.05036	70
4	1.809	0.01366	79
**4-mer Set**	**Erel (kcal/mol)**	**weight**	**degeneracy**
1	0.000	0.67097	13
2	0.192	0.07767	2
3	0.242	0.03695	1
4	0.431	0.05123	2

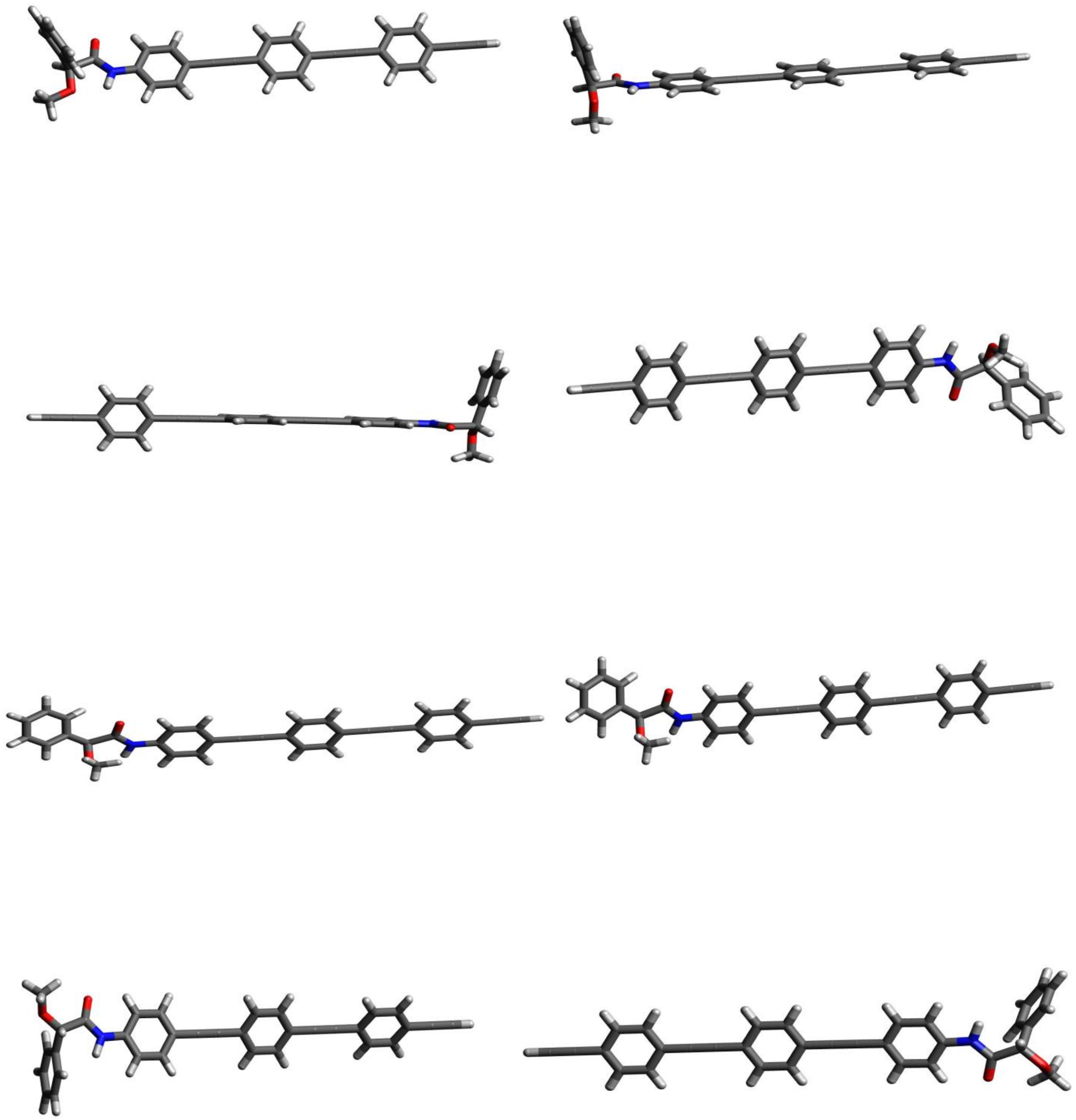

Figure S1. MD conformers obtained for the monomer ordered from lower to higher energy.

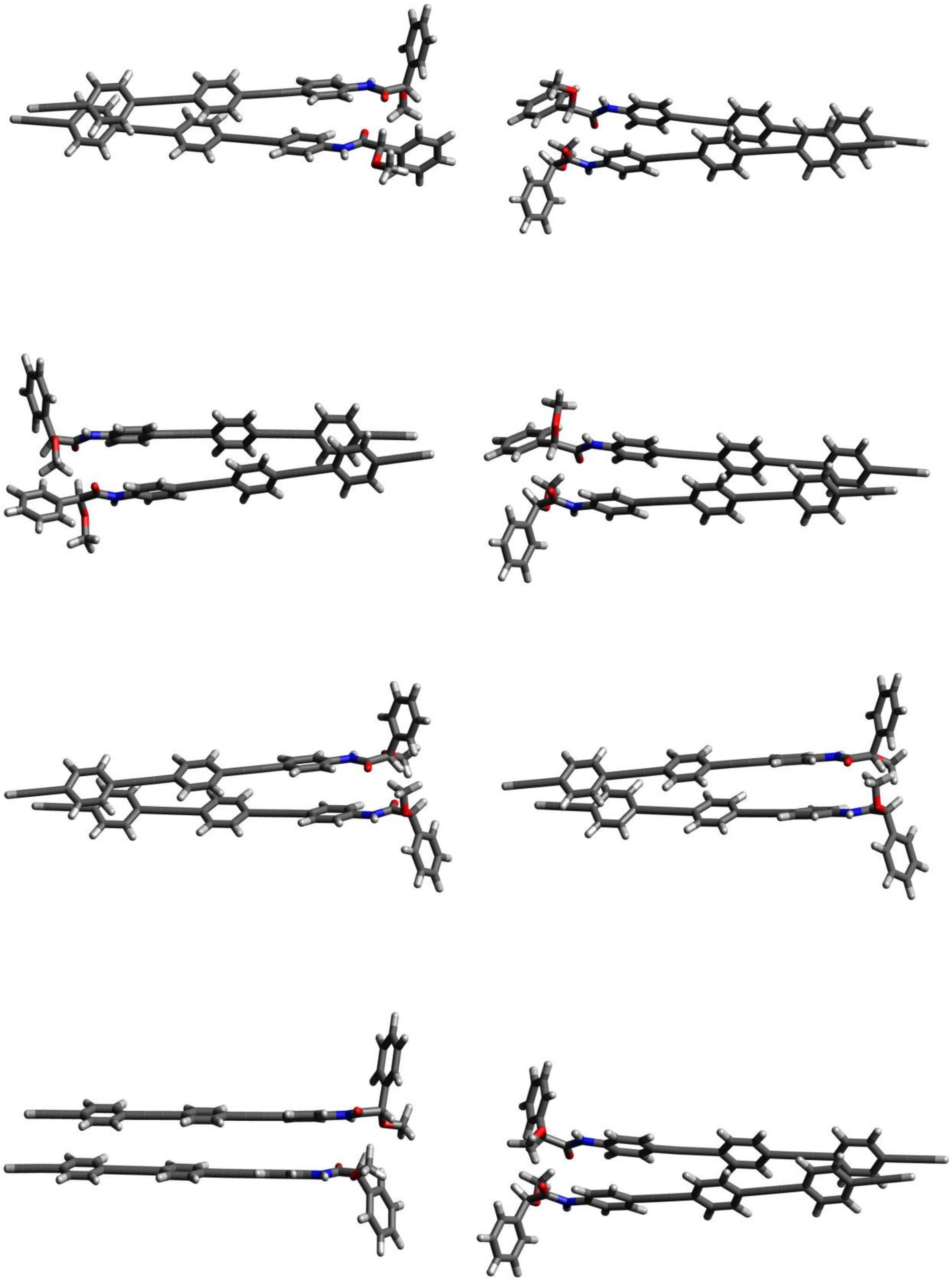

Figure S2. MD four most stable conformers obtained from the dimer hh (left) and ht configurations (right) ordered from lower to higher energy.

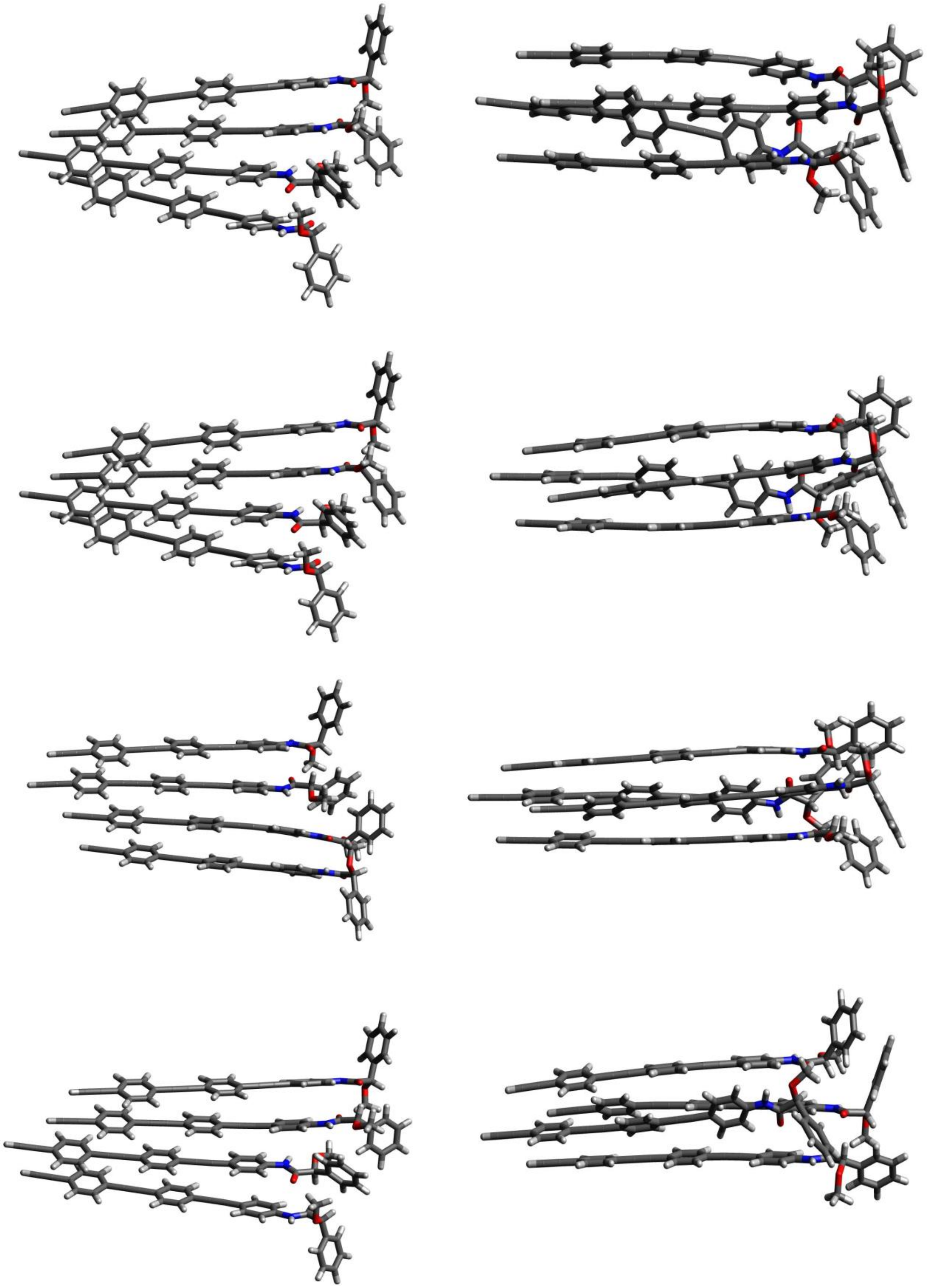

Figure S3. MD four most stable conformers obtained from the **4-mer** hh (left) and ht configurations (right) ordered from lower to higher energy.

2. Table S2: Geometries used in the spectrum calculations. XYZ file format with atom symbol followed by cartesian coordinates in Amgstrom.

Monomer X-Ray geometry obtained from the crystallographic study carried out in Ref. 3 (details are given in the corresponding Supplementary Materials).

N	3.044000	5.988000	5.900000
H	3.856000	6.098000	6.236000
C	1.954000	6.241000	6.671000
O	0.795000	6.164000	6.305000
C	2.270000	6.620000	8.124000
H	1.633000	7.333000	8.416000
O	3.599000	7.091000	8.308000
C	3.781000	8.424000	7.842000
H	3.696000	8.443000	6.866000
H	4.671000	8.741000	8.099000
H	3.099000	9.005000	8.241000
C	2.096000	5.418000	9.024000
C	3.048000	4.406000	9.024000
H	3.787000	4.451000	8.429000
C	2.924000	3.329000	9.891000
H	3.584000	2.645000	9.897000
C	1.835000	3.250000	10.751000
H	1.749000	2.514000	11.346000
C	0.880000	4.244000	10.735000
H	0.129000	4.185000	11.315000
C	1.006000	5.328000	9.882000
H	0.346000	6.011000	9.882000
C	3.014000	5.589000	4.540000
C	2.000000	4.784000	4.037000
H	1.293000	4.500000	4.604000
C	2.021000	4.399000	2.714000
H	1.324000	3.848000	2.376000
C	3.048000	4.807000	1.862000
C	4.064000	5.619000	2.378000
H	4.770000	5.909000	1.811000
C	4.048000	6.004000	3.704000
H	4.743000	6.553000	4.046000
C	3.066000	4.366000	0.494000
C	3.060000	3.948000	-0.623000
C	3.073000	3.464000	-1.975000
C	2.096000	2.572000	-2.436000

H	1.401000	2.290000	-1.853000
C	2.136000	2.099000	-3.733000
H	1.466000	1.498000	-4.035000
C	3.155000	2.496000	-4.606000
C	4.118000	3.403000	-4.150000
H	4.807000	3.693000	-4.736000
C	4.076000	3.877000	-2.859000
H	4.737000	4.493000	-2.564000
C	3.246000	1.936000	-5.931000
C	3.345000	1.446000	-7.004000
H	3.425000	1.053000	-7.865000

Monomer Conformer1 DFT(B3LYP-D3)/6-31G optimized geometry**

C	-3.747414	0.405577	-0.968504
C	-2.361561	0.479356	-0.909017
C	-1.595156	-0.521717	-0.279835
C	-2.273550	-1.614712	0.296563
C	-3.656186	-1.695560	0.241152
C	-4.409550	-0.690660	-0.390048
N	-5.805740	-0.840111	-0.400499
C	-6.750437	-0.036259	-0.972602
O	-6.544363	0.989993	-1.604367
C	-8.201684	-0.511463	-0.741687
C	-8.929506	0.498405	0.135232
C	-9.059590	0.277298	1.509556
C	-9.696532	1.222087	2.315269
C	-10.195556	2.398678	1.754387
C	-10.055384	2.627031	0.383515
C	-9.427016	1.679806	-0.424039
H	-9.301037	1.860927	-1.486826
H	-10.437521	3.542780	-0.057864
H	-10.690216	3.134508	2.381514
H	-9.798719	1.040321	3.381242
H	-8.663782	-0.637966	1.937833
H	-8.660869	-0.526413	-1.741510
O	-8.187060	-1.820933	-0.188252
C	-9.425924	-2.511092	-0.298823
H	-10.228907	-1.982168	0.229004
H	-9.714004	-2.638046	-1.352049
H	-9.281928	-3.493885	0.154267
H	-6.184236	-1.672017	0.036350

H	-4.163662	-2.545151	0.691493
H	-1.703176	-2.396739	0.786520
C	-0.178012	-0.433709	-0.227099
C	1.035951	-0.359819	-0.181549
C	2.452193	-0.271012	-0.130457
C	3.127431	0.823199	-0.712643
C	4.510079	0.909875	-0.663316
C	5.272939	-0.094089	-0.029800
C	4.598354	-1.188143	0.552573
C	3.215708	-1.274813	0.503185
H	2.706067	-2.119975	0.953812
H	5.176259	-1.965108	1.042024
C	6.689516	-0.005403	0.020498
C	7.903379	0.070460	0.063426
C	9.320324	0.158919	0.113224
C	10.082290	-0.844433	0.747902
C	11.465890	-0.757728	0.796124
C	12.137346	0.334521	0.211941
C	11.377958	1.337594	-0.422344
C	9.994311	1.251824	-0.471090
H	9.416511	2.028194	-0.961327
H	11.888234	2.181576	-0.874338
C	13.560543	0.423226	0.261777
C	14.767954	0.498494	0.304040
H	15.830670	0.564846	0.341167
H	12.044165	-1.533849	1.286253
H	9.572469	-1.688598	1.200025
H	5.019691	1.755084	-1.113981
H	2.549498	1.600077	-1.202011
H	-1.853766	1.327204	-1.356950
H	-4.324839	1.178257	-1.455266

Monomer Conformer2 DFT(B3LYP-D3)/6-31G optimized geometry**

C	3.826203	-0.026411	1.225712
C	2.439817	0.050478	1.185293
C	1.700036	-0.510357	0.125272
C	2.405723	-1.159799	-0.907845
C	3.789087	-1.241714	-0.874054
C	4.515104	-0.678271	0.189121
N	5.913803	-0.799458	0.154920
C	6.843808	-0.360550	1.056851

O	6.615475	0.235458	2.100783
C	8.295959	-0.641347	0.601180
C	8.844826	0.582225	-0.120549
C	9.329853	0.494464	-1.428212
C	9.825244	1.632995	-2.067909
C	9.839767	2.861945	-1.408856
C	9.355244	2.950051	-0.101449
C	8.859321	1.817485	0.541156
H	8.467564	1.884279	1.551203
H	9.362358	3.902893	0.419651
H	10.226244	3.745382	-1.908512
H	10.201207	1.555513	-3.084102
H	9.324842	-0.462908	-1.935407
H	8.866922	-0.811875	1.525018
O	8.372394	-1.774573	-0.252785
C	8.303039	-3.013744	0.444568
H	7.358024	-3.119571	0.995327
H	8.368649	-3.802690	-0.307269
H	9.137889	-3.118192	1.150704
H	6.315429	-1.277365	-0.643479
H	4.318080	-1.745084	-1.679674
H	1.856042	-1.597451	-1.734395
C	0.282082	-0.423885	0.096820
C	-0.932438	-0.350269	0.071705
C	-2.349501	-0.262287	0.045419
C	-3.050355	0.389907	1.082470
C	-4.433682	0.476007	1.057227
C	-5.171393	-0.086619	-0.006018
C	-4.471087	-0.738733	-1.043074
C	-3.087736	-0.824812	-1.017813
H	-2.558165	-1.328117	-1.819894
H	-5.029603	-1.174348	-1.864949
C	-6.588644	0.001726	-0.031808
C	-7.803049	0.077627	-0.053655
C	-9.220658	0.166409	-0.078663
C	-9.958451	-0.396556	-1.141108
C	-11.342678	-0.309707	-1.164956
C	-12.038787	0.342689	-0.128163
C	-11.303510	0.905592	0.933749
C	-9.919265	0.819278	0.958533
H	-9.360070	1.255221	1.779624
H	-11.832897	1.409498	1.735498

C	-13.462608	0.431776	-0.153171
C	-14.670542	0.507387	-0.174371
H	-15.733738	0.574038	-0.192984
H	-11.902359	-0.745435	-1.985857
H	-9.429530	-0.900506	-1.943120
H	-4.963256	0.979549	1.859194
H	-2.491811	0.825687	1.904149
H	1.910758	0.554712	1.987245
H	4.383476	0.408021	2.043200

Dimer DFT(B3LYP-D3)/6-31G optimized geometry**

C	4.072734	1.847056	-1.183029
C	2.724141	1.789357	-0.873813
C	2.295698	1.382684	0.406285
C	3.276532	1.044252	1.357399
C	4.632369	1.109988	1.057384
C	5.043180	1.509745	-0.224729
N	6.386543	1.609294	-0.611254
C	7.516765	1.291524	0.073061
O	7.577217	0.906034	1.240322
C	8.801183	1.542512	-0.751050
C	9.348212	2.926505	-0.424370
C	9.644094	3.255239	0.905487
C	10.151395	4.514250	1.220997
C	10.368003	5.458080	0.214007
C	10.073506	5.132272	-1.109752
C	9.565586	3.871428	-1.430735
H	9.342878	3.611052	-2.458524
H	10.239462	5.859908	-1.899196
H	10.763564	6.439050	0.460741
H	10.376692	4.758995	2.255031
H	9.458016	2.525552	1.687124
O	8.561683	1.434831	-2.143987
C	8.562933	0.077500	-2.610955
H	9.547366	-0.383003	-2.459759
H	8.345314	0.122539	-3.680125
H	7.804431	-0.525819	-2.101949
H	9.517348	0.779310	-0.423615
H	6.568336	1.846681	-1.580454
H	5.378066	0.858801	1.797709
H	2.962880	0.722029	2.344617

C	0.907810	1.309631	0.699333
C	-0.292763	1.253215	0.888792
C	-1.699747	1.199971	1.069406
C	-2.558386	1.782210	0.113449
C	-3.933761	1.733829	0.271985
C	-4.506683	1.098623	1.392807
C	-3.649692	0.512421	2.347725
C	-2.272849	0.562487	2.189647
H	-1.621272	0.105380	2.926897
H	-4.081742	0.016337	3.210668
C	-5.918270	1.043065	1.535347
C	-7.131800	1.007171	1.617696
C	-8.549661	0.985012	1.697190
C	-9.325144	1.781599	0.829037
C	-10.709409	1.760982	0.896313
C	-11.369064	0.939818	1.830994
C	-10.597777	0.145188	2.700981
C	-9.211707	0.167226	2.635929
H	-8.623781	-0.449653	3.307533
H	-11.100002	-0.492077	3.421371
C	-12.793865	0.887510	1.863619
C	-14.003202	0.836870	1.864786
H	-15.066912	0.791375	1.842201
H	-11.298245	2.356005	0.207359
H	-8.823782	2.401114	0.093670
H	-4.585753	2.170802	-0.476161
H	-2.126734	2.263898	-0.757361
H	1.983124	2.050188	-1.621893
H	4.385544	2.157475	-2.176742
C	3.188991	-1.770927	-0.905781
C	1.847554	-1.628979	-1.238720
C	0.824029	-2.032828	-0.361108
C	1.192742	-2.599280	0.875883
C	2.527738	-2.743508	1.215074
C	3.539993	-2.326762	0.334045
N	4.866755	-2.484219	0.766164
C	6.019456	-2.028358	0.200245
O	6.100356	-1.436408	-0.871011
C	7.253870	-2.246030	1.095348
C	8.531805	-2.409492	0.303279
C	9.654301	-1.640522	0.623381
C	10.855116	-1.815867	-0.069801

C	10.937405	-2.763252	-1.090411
C	9.818159	-3.538212	-1.412098
C	8.624649	-3.366337	-0.714507
H	7.754305	-3.965159	-0.965189
H	9.878426	-4.275731	-2.207033
H	11.867509	-2.899245	-1.634467
H	11.720096	-1.210441	0.185159
H	9.570554	-0.882597	1.396715
H	7.329131	-1.331888	1.702231
O	6.989505	-3.372622	1.942162
C	7.861301	-3.461086	3.061058
H	8.904521	-3.594346	2.748342
H	7.545454	-4.332213	3.638847
H	7.795811	-2.562402	3.692223
H	5.016702	-2.991516	1.630488
H	2.796088	-3.171179	2.177996
H	0.418066	-2.912351	1.567651
C	-0.548611	-1.858585	-0.679534
C	-1.737401	-1.709004	-0.890570
C	-3.132139	-1.525867	-1.077074
C	-3.639411	-0.830566	-2.194132
C	-5.003476	-0.626927	-2.341402
C	-5.911637	-1.114934	-1.379024
C	-5.404873	-1.819804	-0.267356
C	-4.042673	-2.019653	-0.118881
H	-3.659407	-2.544839	0.749004
H	-6.096639	-2.183771	0.484148
C	-7.309288	-0.900766	-1.508199
C	-8.511976	-0.733731	-1.585257
C	-9.917200	-0.537086	-1.643669
C	-10.479198	0.415908	-2.517801
C	-11.849685	0.631282	-2.541473
C	-12.705600	-0.101569	-1.695550
C	-12.145958	-1.060709	-0.829327
C.	-10.776299	-1.274232	-0.803237
H	-10.349889	-1.996083	-0.115580
H	-12.797626	-1.612900	-0.161991
C	-14.113472	0.128118	-1.713300
C	-15.307969	0.324036	-1.733473
H	-16.359137	0.495219	-1.757329
H	-12.274393	1.370469	-3.212660
H	-9.825249	0.988327	-3.167279

H	-5.384465	-0.085163	-3.200940
H	-2.946717	-0.444370	-2.934434
H	1.579843	-1.181902	-2.190096
H	3.965440	-1.434225	-1.576294

4-mer DFT(B3LYP-D3)/6-31G optimized geometry**

C	6.259178	4.152896	1.488061
C	4.906350	4.455712	1.388770
C	4.304499	4.640591	0.127289
C	5.103673	4.536606	-1.028460
C	6.458034	4.238008	-0.926442
C	7.033709	4.039782	0.331282
N	8.424853	3.715031	0.434682
C	8.895617	2.451982	0.375866
O	8.187354	1.440909	0.322161
C	10.422058	2.318164	0.385453
C	10.903042	1.514474	1.575983
C	10.521271	1.882380	2.872200
C	10.987819	1.165968	3.971735
C	11.856448	0.085968	3.785124
C	12.249379	-0.276407	2.496660
C	11.768036	0.432965	1.394293
H	12.049221	0.134672	0.387918
H	12.921355	-1.115816	2.346071
H	12.222283	-0.470664	4.642660
H	10.676670	1.447186	4.973172
H	9.852122	2.726843	3.012391
H	10.671911	1.779535	-0.537955
O	10.988550	3.631599	0.349822
C	12.391707	3.651726	0.118871
H	12.939890	3.143666	0.920972
H	12.688838	4.701457	0.084703
H	12.640855	3.173237	-0.839377
H	9.123253	4.448573	0.417583
H	7.069801	4.102745	-1.812244
H	4.642466	4.650507	-2.002975
C	2.905142	4.871192	0.033748
C	1.698417	5.013907	-0.018095
C	0.286758	5.162121	-0.071702
C	-0.460631	5.330978	1.111824
C	-1.840874	5.450482	1.065248

C	-2.526246	5.407145	-0.166400
C	-1.776829	5.240863	-1.350222
C	-0.396902	5.120880	-1.304298
H	0.170258	4.975435	-2.217205
H	-2.296987	5.195052	-2.300817
C	-3.941198	5.514802	-0.206796
C	-5.154923	5.599704	-0.223593
C	-6.571925	5.695647	-0.230534
C	-7.289017	5.794506	0.979529
C	-8.672877	5.879014	0.977566
C	-9.389932	5.868357	-0.234224
C	-8.675853	5.772644	-1.444456
C	-7.291105	5.686561	-1.443448
H	-6.747961	5.607790	-2.379357
H	-9.222713	5.757939	-2.381424
C	-10.815173	5.912538	-0.229119
C	-12.025228	5.929016	-0.211496
H	-13.089322	5.905178	-0.175882
H	-9.218236	5.928639	1.913283
H	-6.744159	5.785373	1.917191
H	-2.410299	5.567418	1.980879
H	0.057266	5.357517	2.064805
H	4.296202	4.536127	2.281945
H	6.720369	3.991143	2.457146
C	4.872668	1.254656	-0.527687
C	3.548455	1.384187	-0.141549
C	2.516725	1.411540	-1.100262
C	2.867602	1.258340	-2.454755
C	4.192566	1.123694	-2.853186
C	5.213161	1.153908	-1.886887
N	6.585639	1.170773	-2.212632
C	7.144544	1.015301	-3.443962
O	6.559461	0.617959	-4.448380
C	8.634617	1.386029	-3.558701
C	9.523015	0.160042	-3.432562
C	10.008825	-0.481493	-4.575593
C	10.795126	-1.629199	-4.458159
C	11.108694	-2.134769	-3.195081
C	10.623160	-1.497940	-2.051209
C	9.827603	-0.359806	-2.169897
H	9.407960	0.100766	-1.283309
H	10.827651	-1.905430	-1.066379

H	11.720252	-3.027682	-3.102400
H	11.166363	-2.122729	-5.351766
H	9.763386	-0.086696	-5.557877
H	8.725245	1.795455	-4.574283
O	8.947195	2.398085	-2.599556
C	10.152959	3.090952	-2.896199
H	10.072942	3.635727	-3.847783
H	10.317424	3.798146	-2.081389
H	11.007509	2.403344	-2.957426
H	7.210078	1.404465	-1.443278
H	4.446001	1.014141	-3.897460
H	2.081926	1.247254	-3.202972
C	1.156649	1.572622	-0.726904
C	-0.019504	1.702049	-0.443508
C	-1.401108	1.834489	-0.151122
C	-1.861242	1.949611	1.175988
C	-3.215524	2.071791	1.447714
C	-4.161883	2.084638	0.403390
C	-3.701587	1.968744	-0.924461
C	-2.349223	1.846563	-1.195767
H	-2.003209	1.755344	-2.219810
H	-4.424499	1.973689	-1.732759
C	-5.550933	2.204007	0.665441
C	-6.750232	2.297438	0.846841
C	-8.153700	2.416668	1.018946
C	-9.005594	2.444398	-0.103315
C	-10.376081	2.578381	0.051347
C	-10.945706	2.688192	1.333602
C	-10.097318	2.656714	2.457275
C	-8.724775	2.522498	2.303264
H	-8.076648	2.500063	3.173096
H	-10.530236	2.735134	3.449162
C	-12.358261	2.806466	1.485921
C	-13.560137	2.891800	1.603635
H	-14.620774	2.914765	1.695388
H	-11.024613	2.600342	-0.816196
H	-8.572746	2.365639	-1.094023
H	-3.560964	2.154988	2.472728
H	-1.139606	1.939068	1.985474
H	3.305916	1.465227	0.911235
H	5.658053	1.266302	0.217954
C	3.135206	-1.720374	0.356379

C	1.798474	-1.580736	0.709408
C	0.790188	-1.735834	-0.257941
C	1.156497	-2.015771	-1.590199
C	2.491840	-2.165792	-1.934679
C	3.481418	-2.029394	-0.958246
N	4.850331	-2.193793	-1.322339
C	5.573675	-3.300050	-1.034175
O	5.179024	-4.262661	-0.374381
C	7.007589	-3.278509	-1.608199
C	8.018472	-3.094368	-0.498289
C	8.087770	-1.876668	0.185557
C	9.043062	-1.683881	1.180449
C	9.919068	-2.718663	1.521680
C	9.851952	-3.937082	0.845242
C	8.908670	-4.118835	-0.169096
H	8.863532	-5.062186	-0.706510
H	10.533868	-4.743364	1.100008
H	10.653739	-2.565229	2.305092
H	9.105186	-0.722572	1.677329
H	7.411075	-1.070035	-0.072073
H	7.154349	-4.262722	-2.072431
O	7.182325	-2.235383	-2.564028
C	6.849850	-2.613028	-3.897993
H	5.815165	-2.979148	-3.967355
H	7.531082	-3.394225	-4.262328
H	6.953039	-1.715779	-4.507372
H	5.300506	-1.493208	-1.900488
H	2.779016	-2.394121	-2.956231
H	0.378772	-2.127442	-2.337688
C	-0.586824	-1.625848	0.064388
C	-1.786875	-1.561432	0.244302
C	-3.196710	-1.474007	0.355342
C	-3.985462	-1.524992	-0.812745
C	-5.362502	-1.414914	-0.741646
C	-6.004247	-1.252808	0.502785
C	-5.219372	-1.211325	1.672364
C	-3.838796	-1.320637	1.599961
H	-3.237428	-1.278152	2.501678
H	-5.708876	-1.083090	2.631943
C	-7.416518	-1.140200	0.554264
C	-8.628418	-1.039567	0.540516
C	-10.036986	-0.882316	0.490848

C	-10.803582	-0.822225	1.671518
C	-12.177757	-0.644611	1.611817
C	-12.830538	-0.520755	0.370944
C	-12.066159	-0.576855	-0.809890
C	-10.693706	-0.758106	-0.750747
H	-10.109641	-0.815932	-1.662319
H	-12.566956	-0.504189	-1.768742
C	-14.246884	-0.371589	0.307616
C	-15.451120	-0.265731	0.245113
H	-16.511321	-0.197254	0.173985
H	-12.761807	-0.588421	2.523832
H	-10.303481	-0.902631	2.630503
H	-5.964627	-1.468111	-1.641651
H	-3.495041	-1.653978	-1.771109
H	1.530758	-1.355644	1.735263
H	3.917282	-1.588299	1.092134
C	1.933643	-4.991816	0.360413
C	0.660888	-5.181603	-0.160180
C	-0.479196	-5.054281	0.657396
C	-0.285294	-4.785104	2.026624
C	0.981318	-4.594692	2.557335
C	2.105791	-4.657663	1.715203
N	3.400611	-4.305262	2.142689
C	3.765108	-3.887488	3.390519
O	3.079559	-3.966930	4.407169
C	5.136676	-3.187027	3.466558
C	4.968560	-1.664942	3.437302
C	3.826133	-1.024894	3.937366
C	3.710820	0.364767	3.861776
C	4.733911	1.132300	3.300966
C	5.883047	0.501234	2.821320
C	5.996841	-0.887489	2.890819
H	6.874130	-1.383583	2.502110
H	6.686071	1.078423	2.371446
H	4.634055	2.210938	3.229039
H	2.813811	0.846532	4.240573
H	3.029255	-1.612470	4.378935
H	5.563348	-3.483792	4.437945
O	5.998987	-3.550139	2.405561
C	6.564431	-4.848458	2.534696
H	7.250063	-4.895530	3.392930
H	7.112753	-5.032848	1.612750

H	5.791240	-5.619505	2.651574
H	4.094804	-4.186243	1.401912
H	1.111017	-4.367736	3.604662
H	-1.153925	-4.695288	2.670698
C	-1.807545	-5.110985	0.155572
C	-2.982299	-5.065378	-0.162000
C	-4.370648	-4.940600	-0.430396
C	-4.890115	-4.980721	-1.741248
C	-6.247367	-4.805496	-1.971513
C	-7.138806	-4.586794	-0.900099
C	-6.620228	-4.555724	0.411654
C	-5.266068	-4.728953	0.640961
H	-4.873343	-4.677890	1.650298
H	-7.295402	-4.368092	1.239215
C	-8.528802	-4.393626	-1.119471
C	-9.725693	-4.227405	-1.265369
C	-11.123148	-4.027167	-1.421471
C	-11.686360	-3.832648	-2.699751
C	-13.048701	-3.615727	-2.846945
C	-13.897130	-3.588316	-1.722343
C	-13.335764	-3.782412	-0.444943
C	-11.974145	-3.998524	-0.296999
H	-11.546329	-4.123237	0.691474
H	-13.979699	-3.740912	0.426444
C	-15.298102	-3.369138	-1.879136
C	-16.486922	-3.190984	-2.022039
H	-17.533249	-3.035293	-2.146329
H	-13.473742	-3.466139	-3.834033
H	-11.039297	-3.850454	-3.570529
H	-6.635731	-4.833234	-2.984541
H	-4.212375	-5.140882	-2.573342
H	0.538148	-5.391333	-1.217505
H	2.803492	-5.031457	-0.286622

8-mer geometry

C	8.208000	-40.707000	-60.176000
N	21.736000	-33.242000	-73.755000
O	23.495502	-34.689884	-73.518166
C	9.016000	-40.399000	-61.003000
O	22.956872	-31.997111	-75.799095

C	9.995000	-39.982000	-61.979000
C	9.602000	-39.518000	-63.223000
C	10.530000	-39.058000	-64.129000
C	11.894000	-39.029000	-63.805000
C	12.290000	-39.498000	-62.543000
C	11.361000	-39.975000	-61.649000
C	12.847000	-38.515000	-64.736000
C	13.624000	-38.071000	-65.553000
C	14.524000	-37.513000	-66.512000
C	14.057000	-37.021000	-67.740000
C	14.920000	-36.468000	-68.649000
C	16.291000	-36.397000	-68.375000
C	16.759000	-36.887000	-67.154000
C	15.892000	-37.451000	-66.243000
C	17.194000	-35.858000	-69.351000
C	17.957000	-35.435000	-70.170000
C	18.914000	-34.910000	-71.103000
C	18.515000	-34.360000	-72.324000
C	19.455000	-33.822000	-73.189000
C	20.807000	-33.833000	-72.855000
C	21.216000	-34.380000	-71.656000
C	20.277000	-34.912000	-70.780000
C	22.978327	-33.696677	-74.018985
C	23.792915	-32.831006	-75.003325
C	24.747846	-31.939586	-74.259950
C	24.302030	-30.822273	-73.603209
C	25.186486	-29.970399	-72.962028
C	26.547876	-30.255467	-72.971224
C	27.002864	-31.382587	-73.610973
C	26.116520	-32.230069	-74.255781
C	22.292691	-32.667504	-76.836990
H	7.525000	-40.828000	-59.552000
H	8.680000	-39.519000	-63.454000
H	10.243000	-38.756000	-64.983000
H	13.209000	-39.487000	-62.305000
H	11.645000	-40.301000	-60.802000
H	13.130000	-37.072000	-67.943000
H	14.585000	-36.130000	-69.472000
H	17.685000	-36.832000	-66.949000
H	16.228000	-37.798000	-65.425000
H	17.596000	-34.355000	-72.564000
H	19.175000	-33.444000	-74.015000

H	22.138000	-34.393000	-71.428000
H	20.562000	-35.281000	-69.953000
H	24.315782	-33.433754	-75.607078
H	23.371297	-30.629238	-73.588218
H	24.865208	-29.193830	-72.517969
H	27.160418	-29.673402	-72.538374
H	27.931856	-31.582584	-73.610327
H	26.437937	-33.007983	-74.694923
H	21.493818	-32.379812	-74.155008
H	22.948585	-33.139822	-77.393958
H	21.804984	-32.019618	-77.386342
H	21.662114	-33.315146	-76.456995
C	7.801000	-44.166000	-62.021000
N	24.419000	-37.464000	-72.123000
O	25.964320	-39.079690	-71.622303
C	8.786000	-43.902000	-62.648000
O	26.135929	-36.256706	-73.802026
C	9.977000	-43.540000	-63.380000
C	9.889000	-42.991000	-64.648000
C	11.019000	-42.583000	-65.319000
C	12.285000	-42.696000	-64.725000
C	12.371000	-43.252000	-63.439000
C	11.241000	-43.675000	-62.781000
C	13.449000	-42.237000	-65.413000
C	14.413000	-41.836000	-66.028000
C	15.535000	-41.327000	-66.751000
C	15.374000	-40.744000	-68.017000
C	16.449000	-40.238000	-68.699000
C	17.735000	-40.308000	-68.150000
C	17.899000	-40.889000	-66.890000
C	16.818000	-41.404000	-66.208000
C	18.863000	-39.816000	-68.890000
C	19.811000	-39.435000	-69.512000
C	20.981000	-38.965000	-70.199000
C	20.890000	-38.330000	-71.441000
C	22.030000	-37.849000	-72.065000
C	23.278000	-38.001000	-71.466000
C	23.383000	-38.632000	-70.244000
C	22.243000	-39.108000	-69.608000
C	25.646893	-38.022352	-72.156748
C	26.716438	-37.197909	-72.904003
C	27.568465	-36.430839	-71.931520

C	27.092877	-35.305023	-71.314211
C	27.893670	-34.565876	-70.457682
C	29.199328	-34.977944	-70.208680
C	29.680030	-36.116473	-70.809189
C	28.878427	-36.849347	-71.668165
C	25.646180	-36.820071	-74.989713
H	6.996000	-44.247000	-61.557000
H	9.038000	-42.895000	-65.060000
H	10.942000	-42.221000	-66.194000
H	13.219000	-43.337000	-63.020000
H	11.315000	-44.060000	-61.915000
H	14.508000	-40.698000	-68.405000
H	16.321000	-39.836000	-69.550000
H	18.764000	-40.929000	-66.499000
H	16.948000	-41.814000	-65.361000
H	20.044000	-38.229000	-71.860000
H	21.959000	-37.412000	-72.906000
H	24.234000	-38.741000	-69.835000
H	22.319000	-39.536000	-68.764000
H	27.300747	-37.821870	-73.424278
H	26.198479	-35.025475	-71.475548
H	27.553876	-33.782592	-70.043493
H	29.756165	-34.473732	-69.628175
H	30.568740	-36.403073	-70.632171
H	29.217186	-37.635825	-72.079575
H	24.338563	-36.567357	-72.512386
H	26.360531	-37.327680	-75.429624
H	25.338074	-36.106596	-75.587035
H	24.898216	-37.419471	-74.784737
C	7.608000	-47.621000	-64.005000
N	26.446000	-42.207000	-70.247000
O	27.713455	-43.971926	-69.519123
C	8.719000	-47.432000	-64.409000
O	28.564778	-41.118893	-71.490230
C	10.061000	-47.161000	-64.869000
C	10.280000	-46.573000	-66.103000
C	11.554000	-46.253000	-66.514000
C	12.658000	-46.498000	-65.683000
C	12.432000	-47.094000	-64.433000
C	11.160000	-47.428000	-64.035000
C	13.973000	-46.129000	-66.102000
C	15.072000	-45.802000	-66.492000

C	16.357000	-45.379000	-66.951000
C	16.508000	-44.750000	-68.195000
C	17.739000	-44.327000	-68.624000
C	18.875000	-44.530000	-67.831000
C	18.728000	-45.157000	-66.592000
C	17.491000	-45.590000	-66.165000
C	20.167000	-44.125000	-68.306000
C	21.252000	-43.815000	-68.707000
C	22.572000	-43.437000	-69.123000
C	22.792000	-42.763000	-70.328000
C	24.071000	-42.372000	-70.688000
C	25.153000	-42.655000	-69.858000
C	24.953000	-43.325000	-68.669000
C	23.670000	-43.712000	-68.298000
C	27.602504	-42.875081	-70.058474
C	28.868321	-42.133237	-70.537977
C	29.565556	-41.474653	-69.380148
C	29.070363	-40.325969	-68.820813
C	29.739705	-39.687743	-67.788689
C	30.927032	-40.224329	-67.302047
C	31.421494	-41.385748	-67.843486
C	30.753207	-42.018740	-68.878156
C	28.283259	-41.601948	-72.777068
H	6.722000	-47.640000	-63.716000
H	9.543000	-46.388000	-66.673000
H	11.688000	-45.862000	-67.370000
H	13.166000	-47.268000	-63.857000
H	11.023000	-47.842000	-63.190000
H	15.747000	-44.615000	-68.747000
H	17.822000	-43.892000	-69.465000
H	19.488000	-45.288000	-66.038000
H	17.410000	-46.033000	-65.328000
H	22.061000	-42.573000	-70.904000
H	24.211000	-41.908000	-71.506000
H	25.690000	-43.524000	-68.104000
H	23.536000	-44.168000	-67.476000
H	29.491603	-42.794562	-70.958048
H	28.255276	-39.961775	-69.145882
H	29.389809	-38.887480	-67.416213
H	31.394380	-39.789883	-66.598697
H	32.227821	-41.758003	-67.504842
H	31.101140	-42.821226	-69.247767

H	26.522428	-41.296983	-70.604212
H	29.026192	-42.161844	-73.086659
H	28.164437	-40.848467	-73.391385
H	27.460849	-42.136032	-72.753027
C	7.615000	-50.950000	-65.838000
N	27.674000	-47.114000	-68.304000
O	28.628278	-49.017298	-67.459338
C	8.793000	-50.852000	-66.026000
O	30.069608	-46.184503	-69.086294
C	10.215000	-50.693000	-66.223000
C	10.707000	-50.080000	-67.363000
C	12.057000	-49.868000	-67.522000
C	12.964000	-50.248000	-66.522000
C	12.463000	-50.867000	-65.367000
C	11.115000	-51.094000	-65.222000
C	14.361000	-49.990000	-66.678000
C	15.537000	-49.754000	-66.847000
C	16.916000	-49.437000	-67.045000
C	17.346000	-48.778000	-68.205000
C	18.666000	-48.458000	-68.383000
C	19.616000	-48.798000	-67.412000
C	19.191000	-49.456000	-66.256000
C	17.864000	-49.785000	-66.081000
C	21.004000	-48.499000	-67.626000
C	22.166000	-48.279000	-67.808000
C	23.568000	-48.012000	-67.960000
C	24.062000	-47.316000	-69.068000
C	25.415000	-47.036000	-69.171000
C	26.297000	-47.451000	-68.177000
C	25.826000	-48.145000	-67.082000
C	24.469000	-48.422000	-66.969000
C	28.714006	-47.894879	-67.949411
C	30.105522	-47.258858	-68.151891
C	30.633833	-46.714723	-66.854558
C	30.145316	-45.546457	-66.330528
C	30.666280	-45.014298	-65.161930
C	31.693954	-45.679436	-64.500652
C	32.176808	-46.861516	-65.006846
C	31.657810	-47.387317	-66.178867
C	29.988470	-46.589207	-70.427604
H	6.692000	-50.896000	-65.714000
H	10.105000	-49.804000	-68.045000

H	12.379000	-49.458000	-68.317000
H	13.062000	-51.132000	-64.679000
H	10.791000	-51.526000	-64.439000
H	16.714000	-48.550000	-68.876000
H	18.939000	-48.001000	-69.170000
H	19.823000	-49.679000	-65.582000
H	17.593000	-50.250000	-65.298000
H	23.468000	-47.036000	-69.754000
H	25.741000	-46.556000	-69.924000
H	26.427000	-48.434000	-66.406000
H	24.147000	-48.894000	-66.210000
H	30.735574	-47.960089	-68.489042
H	29.437487	-45.093619	-66.775900
H	30.324521	-44.198603	-64.814129
H	32.059160	-45.318173	-63.701588
H	32.872855	-47.320345	-64.550737
H	31.995656	-48.204380	-66.522690
H	27.892216	-46.201644	-68.590458
H	30.724678	-47.204843	-70.628926
H	30.050109	-45.804869	-71.010839
H	29.132645	-47.043035	-70.580615
C	7.856000	-54.484000	-67.465000
N	28.237000	-51.966000	-66.281000
O	28.899864	-53.918734	-65.283378
C	9.052000	-54.464000	-67.438000
O	30.790683	-51.199564	-66.619568
C	10.494000	-54.397000	-67.376000
C	11.221000	-53.830000	-68.409000
C	12.589000	-53.706000	-68.322000
C	13.275000	-54.133000	-67.176000
C	12.536000	-54.706000	-66.130000
C	11.172000	-54.845000	-66.230000
C	14.691000	-53.967000	-67.078000
C	15.891000	-53.808000	-67.032000
C	17.300000	-53.583000	-66.979000
C	17.974000	-52.965000	-68.042000
C	19.323000	-52.732000	-67.979000
C	20.059000	-53.122000	-66.854000
C	19.391000	-53.738000	-65.793000
C	18.036000	-53.980000	-65.861000
C	21.479000	-52.915000	-66.814000
C	22.666000	-52.771000	-66.785000

C	24.087000	-52.596000	-66.682000
C	24.816000	-51.945000	-67.682000
C	26.180000	-51.753000	-67.540000
C	26.842000	-52.213000	-66.405000
C	26.137000	-52.863000	-65.413000
C	24.767000	-53.052000	-65.545000
C	29.144321	-52.809478	-65.747575
C	30.588014	-52.265925	-65.696154
C	30.909320	-51.742973	-64.323356
C	30.411683	-50.541236	-63.892998
C	30.747841	-50.032896	-62.648710
C	31.594300	-50.756402	-61.815029
C	32.083350	-51.970932	-62.229463
C	31.749093	-52.473969	-63.476251
C	30.924023	-51.611118	-67.954175
H	6.931000	-54.370000	-67.509000
H	10.770000	-53.524000	-69.188000
H	13.074000	-53.326000	-69.046000
H	12.983000	-55.002000	-65.346000
H	10.685000	-55.247000	-65.519000
H	17.488000	-52.704000	-68.816000
H	19.761000	-52.302000	-68.704000
H	19.876000	-53.994000	-65.018000
H	17.599000	-54.418000	-65.140000
H	24.374000	-51.635000	-68.464000
H	26.667000	-51.303000	-68.222000
H	26.588000	-53.183000	-64.640000
H	24.284000	-53.494000	-64.857000
H	31.221762	-53.007869	-65.916242
H	29.826455	-50.048825	-64.457059
H	30.403290	-49.193573	-62.365628
H	31.834087	-50.412008	-60.963104
H	32.654792	-52.469392	-61.656721
H	32.090710	-53.314628	-63.756400
H	28.562655	-51.073329	-66.522944
H	31.642718	-52.274754	-68.021898
H	31.140180	-50.837971	-68.514217
H	30.082468	-52.010655	-68.258425
C	8.258000	-57.897000	-68.977000
N	28.162000	-57.028000	-63.856000
O	28.468204	-59.037168	-62.800086
C	9.425000	-57.972000	-68.721000

O	30.783085	-56.464830	-63.680245
C	10.828000	-58.022000	-68.384000
C	11.781000	-57.503000	-69.243000
C	13.112000	-57.489000	-68.893000
C	13.531000	-57.983000	-67.649000
C	12.562000	-58.508000	-66.780000
C	11.236000	-58.536000	-67.141000
C	14.909000	-57.931000	-67.278000
C	16.087000	-57.869000	-67.000000
C	17.472000	-57.758000	-66.672000
C	18.383000	-57.183000	-67.570000
C	19.708000	-57.059000	-67.245000
C	20.183000	-57.520000	-66.011000
C	19.278000	-58.094000	-65.114000
C	17.947000	-58.225000	-65.445000
C	21.579000	-57.427000	-65.695000
C	22.747000	-57.379000	-65.436000
C	24.130000	-57.320000	-65.059000
C	25.085000	-56.717000	-65.883000
C	26.408000	-56.636000	-65.478000
C	26.801000	-57.161000	-64.250000
C	25.872000	-57.765000	-63.429000
C	24.542000	-57.841000	-63.826000
C	28.881052	-57.946277	-63.180525
C	30.324989	-57.519909	-62.840381
C	30.414741	-57.040422	-61.418195
C	29.937121	-55.807188	-61.060627
C	30.065235	-55.340304	-59.761760
C	30.678079	-56.138614	-58.800636
C	31.143147	-57.383577	-59.145170
C	31.018866	-57.845171	-60.445559
C	31.139420	-56.870042	-64.974367
H	7.370000	-57.709000	-69.193000
H	11.513000	-57.152000	-70.085000
H	13.755000	-57.141000	-69.501000
H	12.826000	-58.847000	-65.933000
H	10.593000	-58.906000	-66.547000
H	18.077000	-56.875000	-68.415000
H	20.310000	-56.657000	-67.861000
H	19.584000	-58.397000	-64.267000
H	17.348000	-58.636000	-64.833000
H	24.827000	-56.363000	-66.726000

H	27.049000	-56.218000	-66.042000
H	26.139000	-58.128000	-62.593000
H	23.903000	-58.252000	-63.254000
H	30.930580	-58.308478	-62.954665
H	29.510435	-55.262964	-61.712694
H	29.737657	-54.480320	-59.528855
H	30.775255	-55.823514	-57.909679
H	31.554022	-57.932852	-58.487401
H	31.342526	-58.707462	-60.676641
H	28.594368	-56.160806	-64.006871
H	31.804370	-57.588412	-64.920442
H	31.517110	-56.110097	-65.463070
H	30.343781	-57.197339	-65.444552
C	8.746000	-61.597000	-70.460000
N	27.365000	-61.728000	-61.717000
O	27.376965	-63.758303	-60.656691
C	9.840000	-61.731000	-69.994000
O	29.930058	-61.292317	-61.046463
C	11.152000	-61.850000	-69.402000
C	12.271000	-61.369000	-70.060000
C	13.513000	-61.422000	-69.469000
C	13.669000	-61.947000	-68.178000
C	12.533000	-62.434000	-67.513000
C	11.298000	-62.395000	-68.115000
C	14.955000	-61.965000	-67.556000
C	16.062000	-61.962000	-67.063000
C	17.366000	-61.920000	-66.482000
C	18.453000	-61.381000	-67.185000
C	19.698000	-61.323000	-66.616000
C	19.913000	-61.818000	-65.324000
C	18.833000	-62.356000	-64.622000
C	17.582000	-62.421000	-65.197000
C	21.229000	-61.795000	-64.753000
C	22.329000	-61.805000	-64.281000
C	23.619000	-61.816000	-63.652000
C	24.737000	-61.251000	-64.274000
C	25.963000	-61.237000	-63.629000
C	26.097000	-61.792000	-62.359000
C	25.004000	-62.358000	-61.736000
C	23.770000	-62.368000	-62.374000
C	27.903489	-62.685384	-60.934572
C	29.276939	-62.331953	-60.324750

C	29.120589	-61.869112	-58.902934
C	28.641988	-60.616886	-58.619027
C	28.547109	-60.168708	-57.311471
C	28.932297	-61.002817	-56.266511
C	29.396455	-62.266707	-56.540351
C	29.495137	-62.710628	-57.848544
C	30.502026	-61.704840	-62.259582
H	7.923000	-61.365000	-70.834000
H	12.181000	-60.998000	-70.930000
H	14.272000	-61.099000	-69.940000
H	12.619000	-62.793000	-66.638000
H	10.539000	-62.739000	-67.658000
H	18.324000	-61.050000	-68.066000
H	20.422000	-60.944000	-67.103000
H	18.961000	-62.682000	-63.739000
H	16.861000	-62.809000	-64.714000
H	24.657000	-60.877000	-65.143000
H	26.717000	-60.845000	-64.056000
H	25.094000	-62.743000	-60.871000
H	23.018000	-62.753000	-61.939000
H	29.855861	-63.148183	-60.337993
H	28.370200	-60.047806	-59.329760
H	28.221200	-59.295137	-57.127939
H	28.876299	-60.700779	-55.368443
H	29.651421	-62.839846	-55.826866
H	29.816057	-63.584525	-58.031067
H	27.856688	-60.881734	-61.769572
H	31.113262	-62.453629	-62.095068
H	30.999580	-60.959077	-62.655929
H	29.795068	-61.989420	-62.874625
C	9.420000	-65.210000	-72.323000
N	25.923000	-66.644000	-60.128000
O	25.581048	-68.663037	-59.102247
C	10.389000	-65.421000	-71.654000
O	28.333556	-66.392364	-58.968217
C	11.549000	-65.632000	-70.819000
C	12.807000	-65.239000	-71.244000
C	13.903000	-65.379000	-70.423000
C	13.766000	-65.905000	-69.130000
C	12.490000	-66.301000	-68.702000
C	11.402000	-66.176000	-69.532000
C	14.902000	-66.012000	-68.270000

C	15.889000	-66.087000	-67.571000
C	17.055000	-66.136000	-66.747000
C	18.295000	-65.684000	-67.221000
C	19.406000	-65.714000	-66.421000
C	19.329000	-66.213000	-65.116000
C	18.096000	-66.665000	-64.640000
C	16.979000	-66.642000	-65.448000
C	20.507000	-66.283000	-64.299000
C	21.490000	-66.370000	-63.622000
C	22.629000	-66.471000	-62.755000
C	23.885000	-65.995000	-63.144000
C	24.960000	-66.066000	-62.272000
C	24.803000	-66.619000	-61.004000
C	23.571000	-67.099000	-60.610000
C	22.487000	-67.023000	-61.476000
C	26.227615	-67.633113	-59.264129
C	27.478796	-67.377416	-58.396041
C	27.083640	-66.895433	-57.027139
C	26.650790	-65.609499	-56.831713
C	26.336522	-65.147040	-55.563840
C	26.448954	-66.000779	-54.470822
C	26.864365	-67.296411	-54.660015
C	27.183364	-67.754569	-55.927535
C	29.099808	-66.852952	-60.049673
H	8.705000	-64.921000	-72.848000
H	12.916000	-64.869000	-72.112000
H	14.761000	-65.116000	-70.735000
H	12.377000	-66.659000	-67.830000
H	10.545000	-66.460000	-69.234000
H	18.363000	-65.352000	-68.109000
H	20.236000	-65.393000	-66.755000
H	18.026000	-66.993000	-63.752000
H	16.152000	-66.972000	-65.118000
H	24.003000	-65.622000	-64.009000
H	25.809000	-65.734000	-62.542000
H	23.463000	-67.482000	-59.747000
H	21.639000	-67.348000	-61.197000
H	27.988695	-68.232823	-58.302922
H	26.564314	-65.026307	-57.576944
H	26.045486	-64.251078	-55.441046
H	26.241468	-65.689529	-53.598907
H	26.934007	-67.882690	-53.915026

H	27.469410	-68.650854	-56.050351
H	26.476736	-65.835864	-60.077426
H	29.610725	-67.643886	-59.775599
H	29.716895	-66.148463	-60.335882
H	28.506190	-67.089254	-60.793278

3. Analysis of the non-covalent intermolecular interactions

In order to analyze the nature of the non-covalent intermolecular interactions, we carried out a calculation on the most stable conformer of the dimer. The NonCovalent Interaction (NCI) index analysis is based on the density ρ and its derivatives. Non-covalent interactions can be identified from the reduced density gradient, s. To assign the different interactions NCIPLOT [4] uses the sign of the second density Hessian eigenvalue, λ_2. In this way, Figure S4 shows the variation of s versus (λ_2 sign) x ρ. This term can characterize the strength of the interaction through ρ and its nature via the λ_2 sign. The colour code used to show the different interaction regions is based on the λ_2 sign, denoting blue strong attractive interactions, green van der Waals interactions and red strong repulsive interactions.

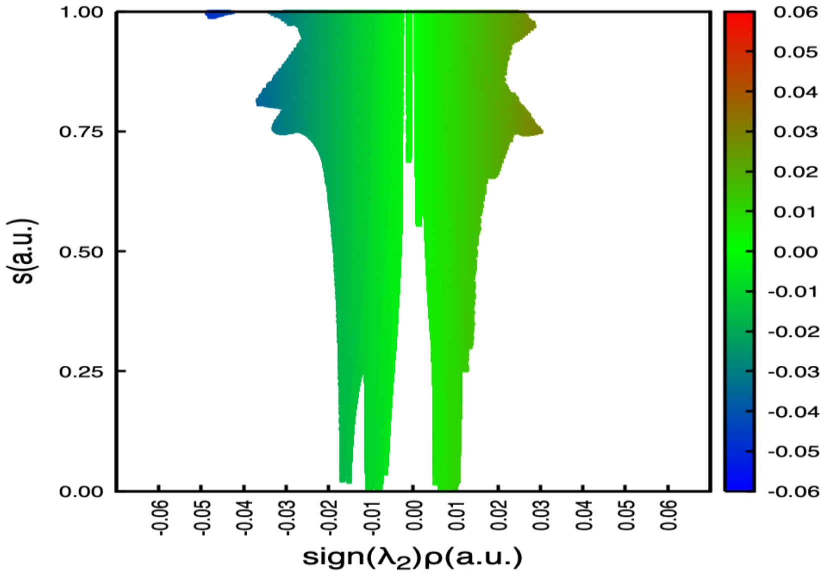

Figure S4. Non-covalent intermolecular interaction in the dimer. s versus sign(λ_2) x ρ plot.

4. Monomer additional calculations.

Due to the almost degeneracy of the two conformers, we additionally carried out the calculations using the 6311++G(d,p) basis set. The results provided similar conformers in terms of the dihedral angles — (O=)C-Cα-O-C(Me) dihedral angle equal to -164º and -78º for Conformer 1 and 2, respectively— and with an energy difference of 0.49 kcal mol^{-1}. The corresponding populations at room temperature are 69.7% for Conformer 1 and 30.3% for Conformer 2. In Figure S5 we compared the ECD spectrum obtained from the 6-31G** optimized geometries to that provided by the 6311++G(d,p) ones.

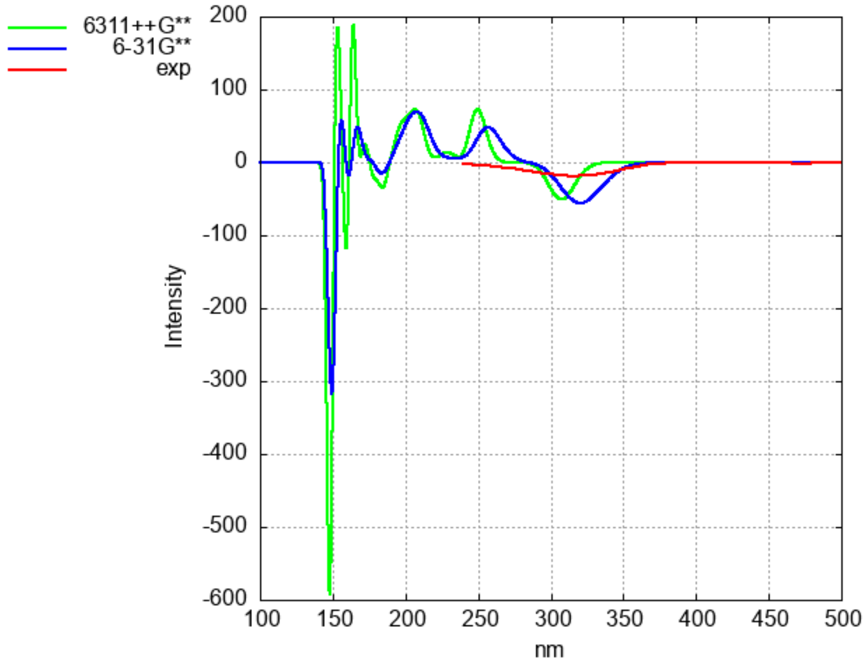

Figure S5. TD-DFT (CAM-B3LYP) ECD spectra obtained from the DFT(B3LYP-D3)/6-31G** and the DFT(B3LYP-D3)/6-311++G** optimized geometries. The corresponding experimental spectrum is plotted for comparison. No correction factors are included in the theoretical wavelengths. Full width at half height (FWHM) equals 20.0 nm and gaussian curves are employed.

Taking into account the possibility of dynamic equilibrium, we additionally evaluated the percentage populations considering the Gibbs free energy of the conformers. From the 6-31G** results, the corresponding values result in 70.2% and 29.8% for Conformer 1 and Conformer 2, respectively; while for the 6-311++G** ones we get 78.9% and 21.1% for Conformer 1 and Conformer 2, respectively. With these two sets of populations we get the spectra and comparison between them and the theoretical ECDs depicted in Figure S5 is provided in Figure S6. From the plots we can conclude that only small differences are observed in the spectrum bands, that are mainly due to intensity changes in the high energy regions.

(a) **(b)**

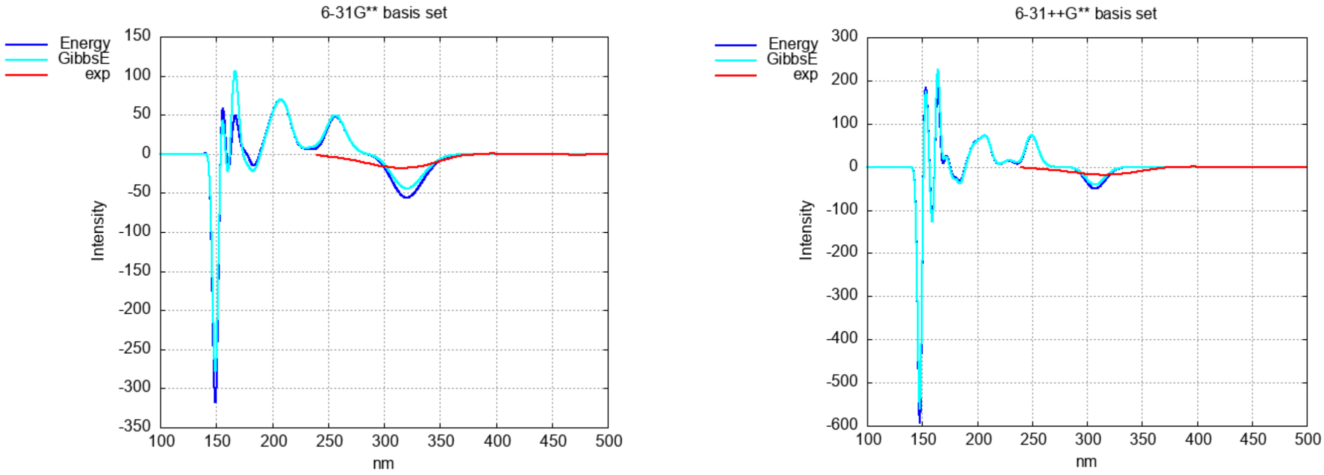

Figure S6. (a) TD-DFT (CAM-B3LYP) ECD spectra obtained from the DFT(B3LYP-D3)/6-31G** and (b) the DFT(B3LYP-D3)/6-311++G** optimized geometries using Gibbs free energy to evaluate the conformer populations. The corresponding experimental spectrum is plotted for comparison. No correction factors are included in the theoretical wavelengths. Full width at half height (FWHM) equals 20.0 nm and gaussian curves are employed.

Table S3. Transitions in the vibronic spectrum for the most stable conformer of oligo(phenyleneethynylene) (OPE) (*S*)-**1**, evaluated within the Franck-Condon approximation and the harmonic oscillator model, with the CAM-B3LYP functional including D3 dispersion and the 6-31G** basis set. Transition energies in wave numbers (cm^{-1}) are relative to the 0-0 (S_0 vibration ground state -> S_1 vibration ground state) energy of 23336.9388 cm^{-1}. Intensities are given in terms of molar absorption coefficients (dm^{3}mol^{-1}cm^{-1}). Notation $|M_1^{v1}; M_2^{v2};\ldots\rangle$ denotes that the vibration excited state of S_1 is a combination of the case where the vibration mode No. M_1 is at v=v_1, the vibration mode No. M_2 is at v=v_2, etc.

**Transition**	**Wavenumber**	**Intensity (x10^5)**
|0> -> |0>	0.0000	5.267
|0> -> |1^1>	7.1894	0.6568
|0> -> |3^1>	13.5259	0.2275
|0> -> |14^1>	104.8558	0.5070
|0> -> |161^1>	122.2447	0.3637
|0> -> |99^1>	1156.9967	0.2294
|0> -> |100^1>	1159.488	0.4369
|0> -> |101^1>	1161.0740	0.4363
|0> -> |144^1>	1672.0796	1.182
|0> -> |148^1>	2248.4347	1.327
|0> -> |148^1;144^1>	3920.5143	0.3221

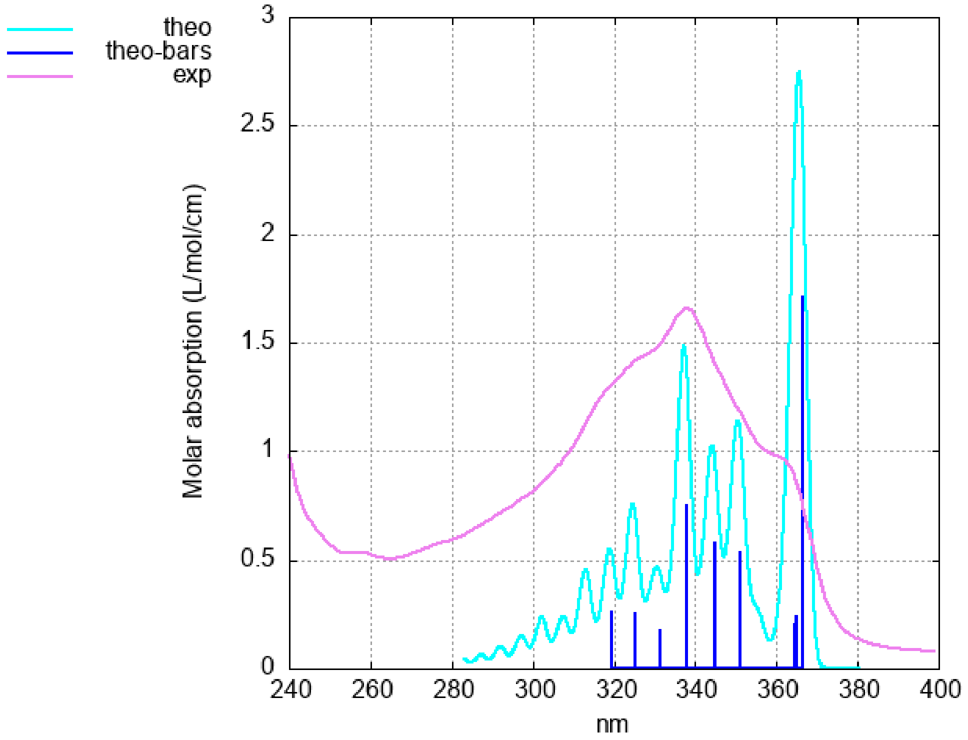

Figure S7. Vibronic spectrum for the most stable conformer of oligo(phenyleneethynylene) (OPE) (*S*)-**1**, evaluated at the CAM-B3LYP-D3/6-31G** level of theory (theo). The bar (theo-bars) and the experimental (exp) spectra are also plotted. The band broadening is simulated by means of Gaussian functions with Half-Width at Half-Maximum of 135.00 cm^{-1}. No correction factors are included in the theoretical wavelengths.

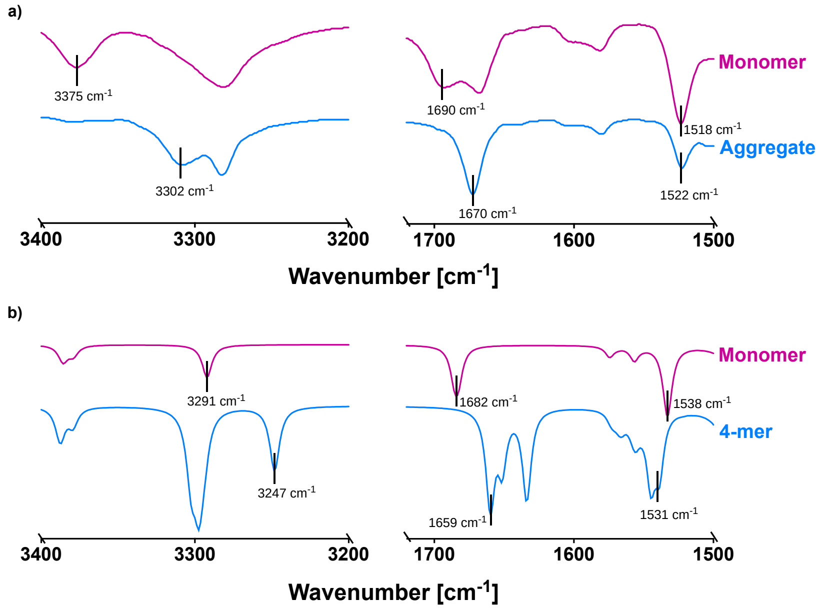

Figure S8. (**a**) Comparison of the experimental IR spectra for the monomer and the aggregate showing the shifts adscribed to the presence of a H-bond network when the later is formed. (**b**) Theoretical IR spectra for the monomer and the **4-mer** evaluated at the corresponding DFT(B3LYP-D3)/6-31G** optimized structures. The calculations were carried out with the B3LYP functional including the D3 dispersion term and the 6-31G** basis set. Lorentzian curves have been used, HWHM equals 8 cm^{-1}. A correction factor of 0.944 has been used for the theoretical wavenumber.

REFERENCES

1. Grimme, S. Conformer-Rotamer Ensemble Sampling Tool (CREST). *J. Chem. Theor. Comput.* **2019**, *15*, 2847–2862.
2. Bannwarth, C.; Caldeweyher, E.; Ehlert, S.; Hansen, A.; Pracht, P.; Seibert, J.; Spicher, S.; Grimme, S. Extended tight-binding quantum chemistry methods, WIREs Computational Molecular Science, **2021**, *11*: e1493.
3. Fernández, Z.; Fernández, B.; Quiñoá, E.; Freire, F. Chiral information harvesting in helical poly(acetylene) derivatives using oligo(*p*-phenyleneethynylene)s as spacers, *Chem. Sci.* **2020**, *11*, 7182–7187.
4. Johnson, E. R.; Keinan, S.; Mori-Sánchez, P.; Contreras-García, J.; Cohen, A. J.; Yang, W. Revealing Noncovalent Interactions, *J. Am. Chem. Soc.* **2010**, *132*, 6498–6506.
